# Towards Practical Application of Li–S Battery with High Sulfur Loading and Lean Electrolyte: Will Carbon-Based Hosts Win This Race?

**DOI:** 10.1007/s40820-023-01120-7

**Published:** 2023-06-07

**Authors:** Yi Gong, Jing Li, Kai Yang, Shaoyin Li, Ming Xu, Guangpeng Zhang, Yan Shi, Qiong Cai, Huanxin Li, Yunlong Zhao

**Affiliations:** 1 Advanced Technology Institute, University of Surrey, Guildford, Surrey , GU2 7XH UK; 2 Department of Chemical and Process Engineering, University of Surrey, Guildford, GU2 7XH UK; 3 Department of Chemistry, Physical and Theoretical Chemistry Laboratory, University of Oxford, Oxford, OX1 3QZ UK; 4 Department of Engineering, University of Cambridge, 9 JJ Thomson Avenue, Cambridge, CB3 0FA UK; 5 State Key Laboratory for Chemo/Biosensing and Chemometrics, College of Chemistry and Chemical Engineering, Hunan University, Changsha, 410082 Hunan People’s Republic of China; 6 College of Materials and Metallurgy, Guizhou University, Guiyang, 550025 People’s Republic of China

**Keywords:** Li–S batteries, Carbon materials, Structural design, Functional modification, Machine learning

## Abstract

As the need for high-energy–density batteries continues to grow, lithium-sulfur (Li–S) batteries have become a highly promising next-generation energy solution due to their low cost and exceptional energy density compared to commercially available Li-ion batteries. Research into carbon-based sulfur hosts for Li–S batteries has been ongoing for over two decades, leading to a significant number of publications and patents. However, the commercialization of Li–S batteries has yet to be realized. This can be attributed, in part, to the instability of the Li metal anode. However, even when considering just the cathode side, there is still no consensus on whether carbon-based hosts will prove to be the best sulfur hosts for the industrialization of Li–S batteries. Recently, there has been controversy surrounding the use of carbon-based materials as the ideal sulfur hosts for practical applications of Li–S batteries under high sulfur loading and lean electrolyte conditions. To address this question, it is important to review the results of research into carbon-based hosts, assess their strengths and weaknesses, and provide a clear perspective. This review systematically evaluates the merits and mechanisms of various strategies for developing carbon-based host materials for high sulfur loading and lean electrolyte conditions. The review covers structural design and functional optimization strategies in detail, providing a comprehensive understanding of the development of sulfur hosts. The review also describes the use of efficient machine learning methods for investigating Li–S batteries. Finally, the outlook section lists and discusses current trends, challenges, and uncertainties surrounding carbon-based hosts, and concludes by presenting our standpoint and perspective on the subject.

## Highlights

- A comprehensive discussion of the approaches for developing carbon-based sulfur hosts is presented, encompassing structural design and functional optimization.
- The recent implementation of effective machine learning methods in discovering carbon-based sulfur hosts has been systematically examined.
- The challenges and future directions of carbon-based sulfur hosts for practically application have been comprehensively discussed.
- A summary of the strengths and weaknesses, along with the outlook on carbon-based sulfur hosts for practical application has been incorporated.

## Graphical abstract

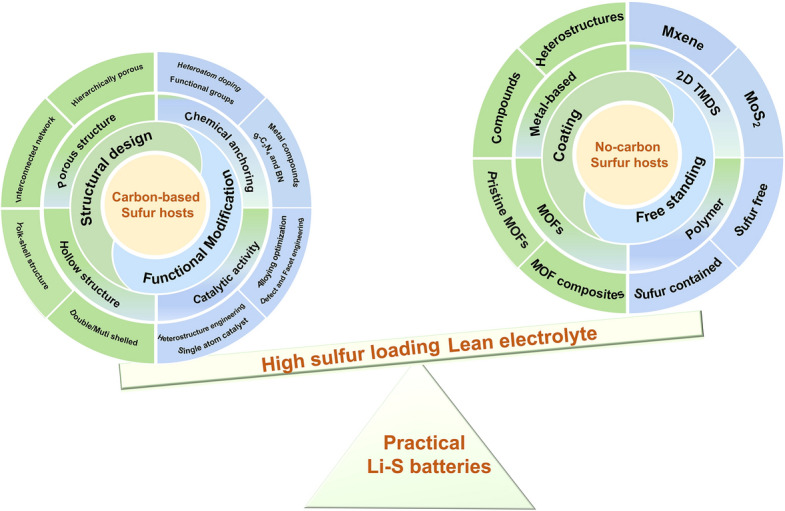

## Introduction

**Fig. 1:**
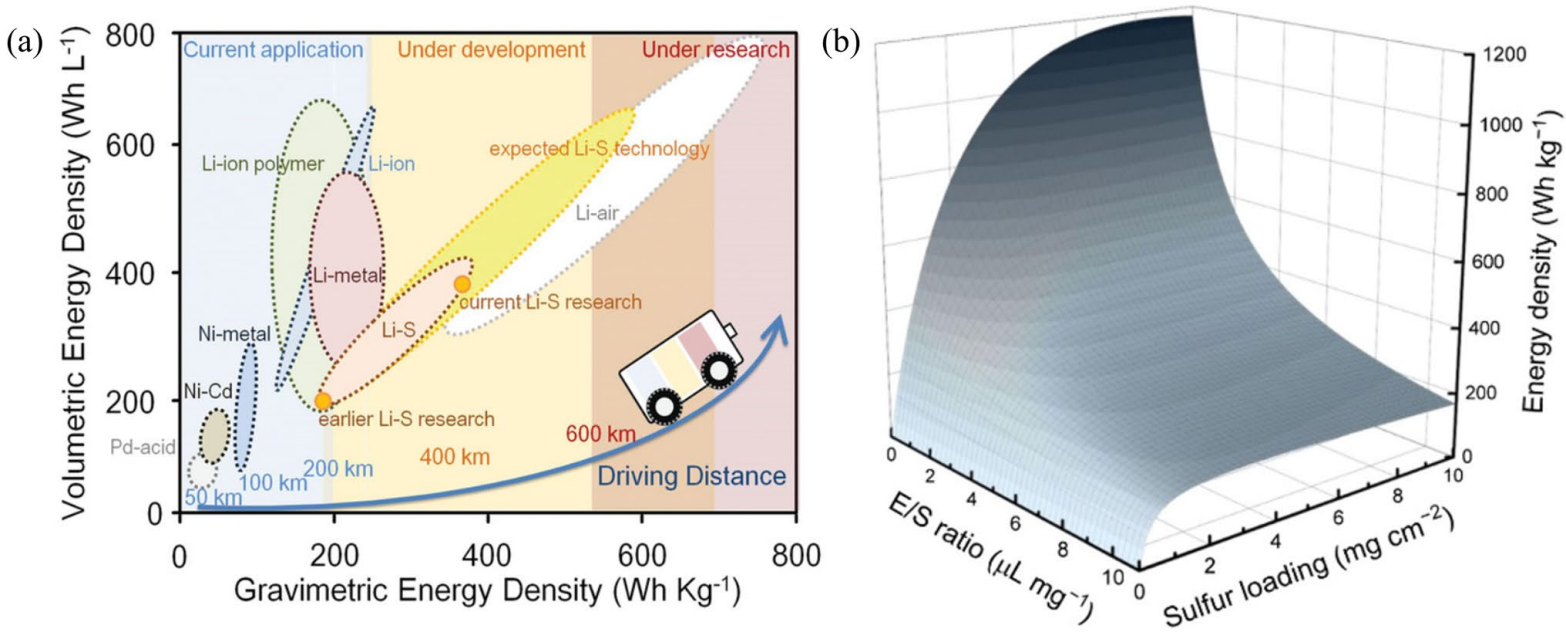
Reproduced with permission from Ref. [22]. Copyright 2019 John Wiley and Sons; **a** Status of different types of batteries. Reproduced with permission from Ref. [8]. Copyright 2020 American Chemical Society; **b** 3D diagram of calculated gravitic gravimetric energy densities of Li–S pouch cells based on sulfur loading and E/S ratio

The escalating reliance and need for power sources with high energy density have stimulated the advancement of cutting-edge energy storage systems. Currently, the Li-ion battery dominates the market of portable energy storage due to the high reliability and maturity of battery-assembly techniques. Despite the fact that commercialized Li-ion batteries based on insertion chemistry are approaching the theoretical energy density, they may not be adequate for high energy density devices such as electric vehicles, smart grids, and drones, thus prompting the rapid development of energy storage systems with even higher energy density [1], including silicon-based anode and Li anode Li-ion batteries, Li–S and Li-air batteries (Fig. 1a) [2–6]. Among them, Li–S batteries are one of the most potentially commercialized high-capacity energy storage devices due to their low cost, the abundance of sulfur, higher theoretical specific capacity (1,675 mAhg^–1^) and overwhelming energy density (2,600 Wh kg^−1^) which endow them with extremely high potential to realize battery energy density beyond 500 Wh kg^–1^ [7–9].

Despite the attractive features of Li–S batteries, there are still some issues that pose a challenge to their development, such as the "shuttle effect" of LiPSs, slow kinetics of conversion reactions, and the growth of lithium dendrites. The Li–S batteries are still under development since it is still challenging to effectively overcome all the problems at the same time. Based on the Li–S chemistry, various effective strategies have been discovered to solve these problems. Although remarkable progress has been made in Li–S batteries at the laboratory level, such as an ultrastable cycle of over 2,000 cycles and a high specific capacity of over 1,500 mAh g^–1^ [10–12], most of the excellent performances are achieved with low sulfur loading (around 1 mg cm^−2^), high electrolyte intake (over 10 μL mg_S_^–1^), and excessive lithium anode, leading to a substantial sacrifice in overall cell energy density [8, 13]. The relationship between the calculated pouch cell energy density and sulfur loading or E/S ratio is shown in Fig. 1b, which demonstrates that energy density increases significantly as the E/S ratio decreases or sulfur loading increases. To achieve a target energy density of 500 Wh kg^–1^, the sulfur loading and E/S ratio are expected to be 6.0 mg cm^–2^ and 2.5 μL mg_S_^–1^. However, issues with Li–S batteries can exacerbate with high sulfur loading and lean electrolyte. Although highly solvating electrolytes (HSEs) and sparingly solvating electrolytes (SSEs) have emerged as two effective solutions for reducing the E/S ratio and hence increasing the overall practical energy density of batteries, they each face distinct challenges [14]. Therefore, it is crucial to develop versatile cathode materials with innovative structures and functions that can strike a balance between high sulfur loading and low electrolyte. Carbon-based sulfur host materials have made significant progress and are playing an increasingly vital role in practical Li–S batteries [15–17].

Recently, some non-carbon sulfur hosts, such as Nb_4_N_5_–Nb_2_O_5_ nanosheets [18], 2D transition metal carbides/nitrides [19], MoS_2_ [20] poly(aniline) [21] have been developed and showed excellent performance under high sulfur loading and lean electrolyte conditions. Therefore, there is currently a debate about whether carbon-based materials will be the most ideal sulfur hosts for the practical application of Li–S batteries under high sulfur loading and lean electrolyte conditions. Therefore, a comprehensive and detailed review of the systematic development of carbon-based materials sulfur hosts is provided to provide a better understanding of how to create a sulfur host. The raised question of "Will Carbon-Based Hosts Win This Race?" is also discussed.

In this review, the effective strategies for developing carbon-based sulfur hosts have been reviewed and systematically discussed. The reaction mechanism and existing problems of Li–S batteries are first elucidated to clarify the desired properties of cathode materials. The methods used to overcome these problems are categorized into structural design and functional modification and discussed in the following part. The merits of different structures of carbon materials ranging from 0 to 3D as sulfur hosts are discussed. Based on these structures, the functions (chemical anchoring and electrocatalysis) have been introduced into carbon materials to suppress the shuttle effect and boost the conversion of sulfur. The host materials can be modified to interact with LiPSs via polar-polar bonding and Lewis-acid base interaction. To introduce catalytic sites into the carbon materials, heterostructure engineering, alloy optimization, defect manipulating, facet engineering and single-atom tailoring have been developed. Furthermore, the emerging machine learning (ML) method which has been developed in the development of cathode materials is illustrated afterwards. This review provides a comprehensive and detailed understanding of how to develop a sulfur host and is expected to offer instructions for the commercial development of host materials. In addition, the tendency, challenges and merits of carbon-based hosts are sorted out and our standpoint and perspective are proposed at the end of the review.

## Electrochemistry of Li–S Batteries

### Mechanism of Li–S Batteries

**Fig. 2:**
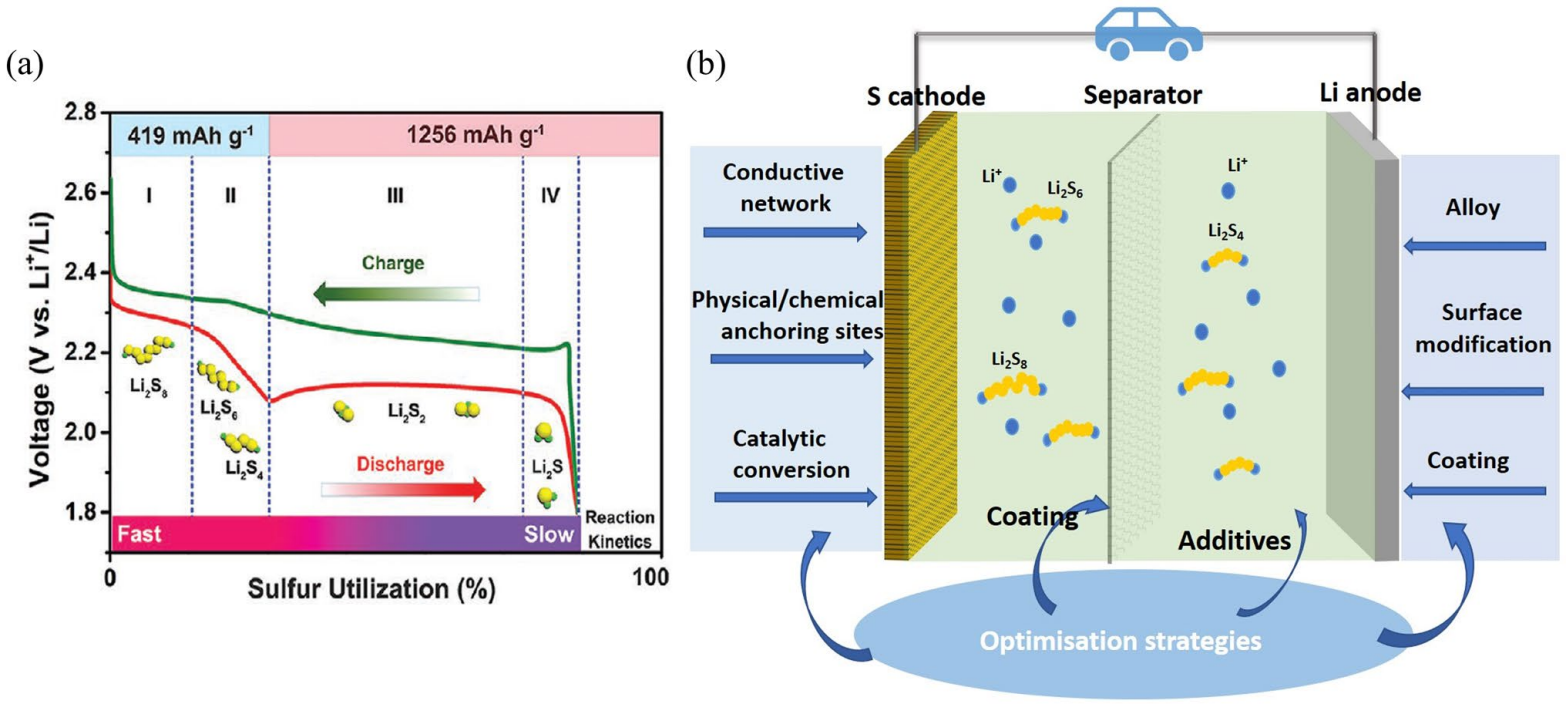
Reproduced with permission from Ref [23]. Copyright 2020 John Wiley and Sons; **a** Typical charge/discharge curves of Li–S batteries (including the various sulfur species). **b** Configuration of Li–S batteries and the recent optimisation strategies for Li–S batteries

The Li–S battery configuration, as illustrated in Fig. 2b, consists of a metallic lithium anode that provides a theoretical capacity of 3,842 mAh g^−1^, and an elemental sulfur cathode that boasts a high theoretical capacity of 1,672 mAh g^−1^, owing to the multi-electron-transfer cathode reaction. This property makes the Li–S battery an appealing energy storage system for achieving full cells with high energy density. Even though the average potential of a Li–S battery is 2.15 V (vs Li^0^/Li^+^) which is lower than that of graphite–LiMO_2_ batteries (> 3 V) [24], its theoretical energy density can reach a very high value of 2,576 wh kg^−1^ [4].

The electrochemical process is illustrated in Fig. 2a, during discharge, the sulfur reacts with Li^+^ converting to polysulfides and eventually to Li_2_S, and conversely, the Li_2_S will decompose into Li^+^ and S during charging. Hence, the overall reaction at the cathode can be represented by Eq. (1):

1 $${\mathrm{S}}_{8}+16 {\mathrm{Li}}^{+}+16 {\mathrm{e}}^{-} \to 8 {\mathrm{Li}}_{2}\mathrm{S}$$

The multi-electron redox reaction of the cathode is complex and the reduction of S can be divided into four stages.

Stage 1: The sulfur is firstly reduced to highly soluble long-chain Li_2_S_8_, which shows a small sloping plateau at about 2.3 V, corresponding to a solid–liquid two-phase reaction in Eq. (2) [25].

2 $${\mathrm{S}}_{8}+2 {\mathrm{Li}}^{+}+2 {e}^{-} \to {\mathrm{Li}}_{2}{\mathrm{S}}_{8}$$

Stage 2: The long-chain Li_2_S_8_ is incrementally reduced to into soluble Li_2_S_4_ by 2 e^−^ in each step, related to a liquid–liquid single-phase reaction shown in Eqs. (3) and (4):

3 $${3\mathrm{Li}}_{2}{\mathrm{S}}_{8}+2{\mathrm{Li}}^{+}+2 {e}^{- }\to 4{\mathrm{Li}}_{2}{\mathrm{S}}_{6}$$

4 $${2\mathrm{Li}}_{2}{\mathrm{S}}_{6}+2{\mathrm{Li}}^{+}+2 {e}^{- }\to 3{\mathrm{Li}}_{2}{\mathrm{S}}_{4}$$

The redox reactions correspond to the plateau at 2.1–2.3 V, during which the concentration and viscosity of soluble polysulfides increase gradually. It is common to see a small voltage peak at the end of this step, which is a result of a higher overpotential caused by the increased electrolyte viscosity [26]. Stages 1 and 2 deliver a quarter (419 mAh g^−1^) of the overall theoretical specific capacity.

Stage 3: Liquid–solid conversions (Eqs. (5) and (6)) occur in this stage, in which the Li_2_S_4_ is reduced to insoluble Li_2_S_2_ and Li_2_S species simultaneously at a long plateau of about 2.1 V.

5 $${\mathrm{Li}}_{2}{\mathrm{S}}_{4}+2{\mathrm{Li}}^{+}+2 {e}^{- }\to 2{\mathrm{Li}}_{2}{\mathrm{S}}_{2}$$

6 $${\mathrm{Li}}_{2}{\mathrm{S}}_{4}+6{\mathrm{Li}}^{+}+6 {e}^{- }\to {\mathrm{Li}}_{2}\mathrm{S}$$

Stage 4: The final stage is the slowest solid–solid conversion shown in Eq. (7):

7 $${\mathrm{Li}}_{2}{\mathrm{S}}_{2}+2{\mathrm{Li}}^{+}+2 {e}^{- }\to {2\mathrm{Li}}_{2}\mathrm{S}$$

Stages 3 and 4 contribute to three fourth (1,256 mAh g^−1^) of the specific capacity. Note that stage 4 surfers from a high polarisation and slow kinetics due to the solid–solid conversion reaction, thereby leading to a final mixture product with Li_2_S_2_ and Li_2_S which is responsible for a real cathode capacity lower than the theoretical value of 1,675 mAh g^−1^ [27].

In terms of the charging process, the solid-state Li_2_S and Li_2_S_2_ species are firstly oxidised into short-chain polysulfides and then into long-chain polysulfides, eventually forming solid S_8_. A small peak is normally seen during the initial stage of the transition of Li_2_S and Li_2_S_2_ to soluble polysulfides, which can be attributed to the potential barrier caused by the phase nucleation of polysulfides [27].

### Challenges in the Li–S Batteries and Their Development Process

Even though the Li–S batteries show many attractive features, the complex electrochemical process involved poses some challenging difficulties. These include: (1) Intrinsic problems of sulfur such as volume expansion and poor electrical conductivity. The lithiation reaction of S_8_ to form Li_2_S results in approximately 80% volume expansion during discharge, which can lead to catastrophic damage to the active materials. The low ionic and electrical conductivities of sulfur and solid Li_2_S_2_/Li_2_S also hinder the full utilization of active materials. (2) Shuttling effect of lithium polysulfides (LiPSs): The intermediate products, soluble lithium polysulfides, can dissolve in the electrolyte during the charge/discharge process and diffuse between the cathode and anode under the force derived from concentration and electric field gradient. This “shuttle-effect” leads to the loss of active materials, passivation of Li anode surface and internal self-discharge, thereby resulting in poor cycling performance [28, 29].. (3) Sluggish conversion reaction kinetics: The reduction of S8 to long-chain LiPSs and the solid conversion of Li_2_S_2_ to Li_2_S at the end of the discharge process is a complex and sluggish step, which limits the wide application of Li–S batteries [30]. (4) Problems with the Li anode including safety issues of metallic Li, rapid Li corrosion in the organic electrolyte, and dendrite growth during the charging process, which can lead to inter short circuits [30, 31]. The metallic Li is also likely to react with diffused soluble LiPSs and passivate the anode surface, resulting in the formation of "dead Li" and deteriorating the cycling performance and coulombic efficiency.

To achieve the commercialization of Li–S batteries, most of these problems must be resolved. Various methods have been developed to address these challenges, as depicted in Fig. 2b, including: (1) incorporation of a conductive network in the cathode to facilitate electron transfer, (2) optimization of cathode structure to accommodate more sulfur and buffer volume expansion, (3) enhancement of physical adsorption and chemical bonding sites to anchor LiPSs, (4) development of redox mediators to catalyze the conversion of sulfur to Li_2_S, (5) implementation of different methods, such as alloying, surface modification, and coating, to protect the anode and minimize Li dendrite growth, (6) coating an effective layer on the separator to inhibit the "shuttle effect," and (7) addition of suitable electrolyte additives to improve the interface between electrodes and electrolyte to inhibit Li dendrite formation, or preparation of high-performance solid electrolytes to address dendrite piercing and diffusion of LiPSs [32]. Recently, modifying the electrolyte has emerged as a highly effective method for enhancing the reaction kinetics between S_8_ and Li_2_S, particularly advantageous under lean electrolyte conditions [33]. For example, Zhang et al. first demonstrated LiPSs exhibit a strong tendency to bond extra lithium ions and form cationic LiPSs which are more sluggish in cathode reactions. To combat this issue, decreasing the salt concentration of the electrolyte has been proven to be an effective way to inhibit the formation of cationic LiPSs, resulting in improved performance of high-energy–density Li–S pouch cells [34]. In addition, adding appropriate redox mediators (RMs) [35] or free radicals [36]in the electrolyte has been proven to be effective to accelerate the reaction kinetics, thereby improving the sulfur utilization and cycling stability.

The capacity and cycling stability of Li–S batteries heavily depend on the performance of the cathode. Although addressing all the challenges of the cathode in Li–S batteries is a daunting task, dedicated efforts by researchers from both industry and academia have led to remarkable findings. Among various materials used for hosting sulfur, carbon-based materials are considered highly promising due to their exceptional electrical conductivity that enhances electron transfer, high specific surface area that accommodates more sulfur and anchors LiPSs, and superior manipulability that facilitates the addition of more effective active sites.

**Fig. 3:**
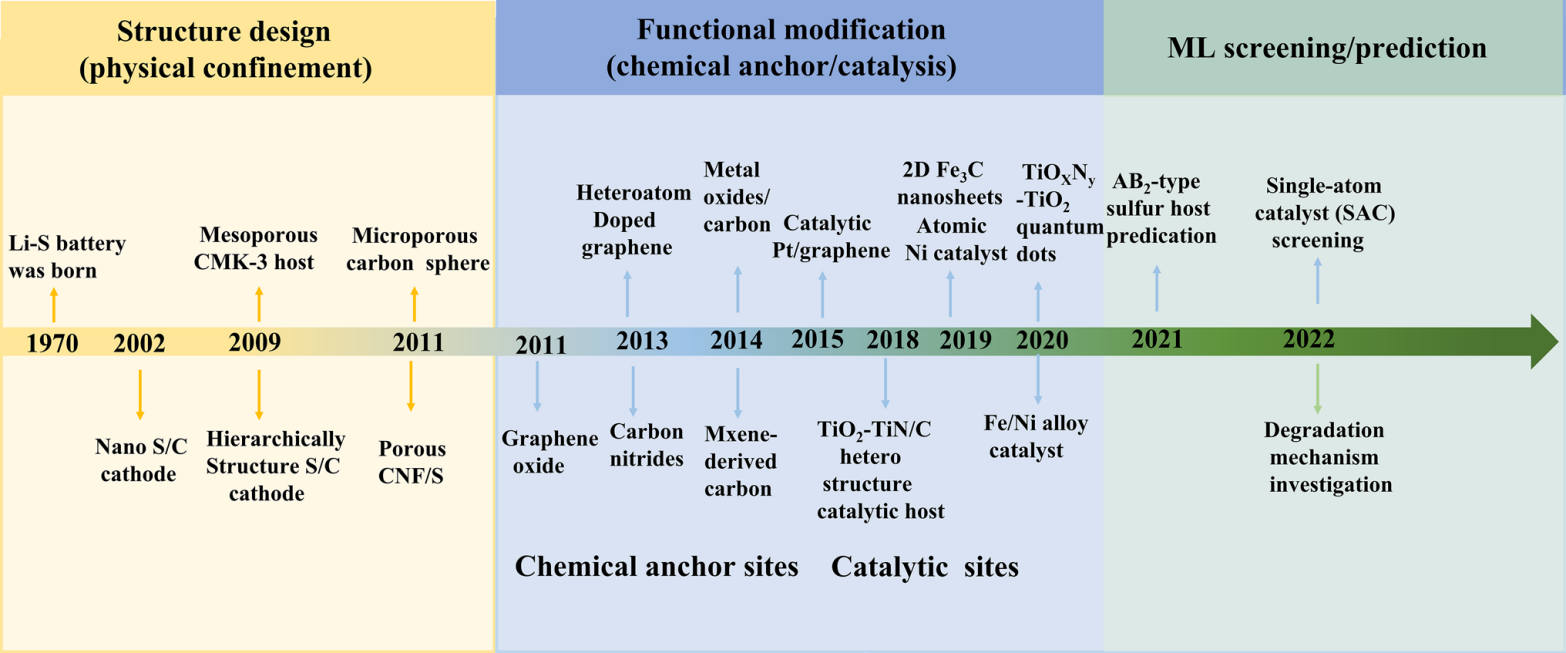
A brief of the timeline and presentative work in the development of cathode materials for Li–S batteries

The development of cathode materials for Li–S batteries can be divided into three distinct stages, as depicted in Fig. 3. The first breakthrough occurred in the 2010s when researchers focused on developing various carbon materials with diverse structures to physically constrain sulfur. In the following decade, with a deeper understanding of Li–S chemistry, bifunctional or multifunctional electrode materials were designed to mitigate the shuttle effect and enhance the conversion of LiPSs. Greater emphasis was placed on the chemical anchoring groups and catalytic sites on carbon materials. More recently, cathode material development has entered a new stage, where machine learning methods are being used to identify promising sulfur hosts to improve efficiency and reduce the costs of trial and error.

## Structural Design for Carbon Materials

### Overview of Strategies for Structural Design

To address the intrinsic issues associated with sulfur, carbon-based nanomaterials that exhibit excellent conductivity and high specific surface area have been identified as a promising solution. These materials can serve as effective hosts for sulfur, facilitating rapid electron/ion transfer channels and preventing polysulfide diffusion between electrodes through physical adsorption within the carbon matrix. Based on their dimensions, carbon-based nanomaterials can be classified into four categories: 0D (e.g., carbon nanospheres), 1D (e.g., carbon nanotubes), 2D (e.g., graphene), and 3D (e.g., carbon networks). Leveraging these fundamental carbon nanomaterials, several strategies have been developed to enhance their host material performance.

Designing appropriate porous structures in fundamental carbon materials is an effective approach for enhancing sulfur utilization in carbon-based host materials. Porous carbon/sulfur composites usually can effectively hold sulfur, thereby increasing more electron pathways, and at the same time, the diffusion of soluble LiPSs is more likely to be physically impediment. Therefore, reasonable design of the structure of host materials is of great importance. So far, a variety of carbon materials with porous structures and high surface area showing excellent performance have been developed. According to the size of the pores or channels, they can be divided into microporous carbon (d < 2 nm), mesoporous carbon (2 nm < d < 50 nm), and microporous (d > 50 nm) [37]. Apart from these materials with different pores, carbon materials with hollow structures exhibit some superiorities as well. It is challenging to determine the superior porous structure as small pores offer high surface area and close contact, but low sulfur loading, whereas large pores allow for high sulfur loading, but with relatively low sulfur utilization. To address this issue, distinguishing between outer and inner surfaces should be considered based on the underlying materials [38].

### 0D Carbon Materials

**Fig. 4:**
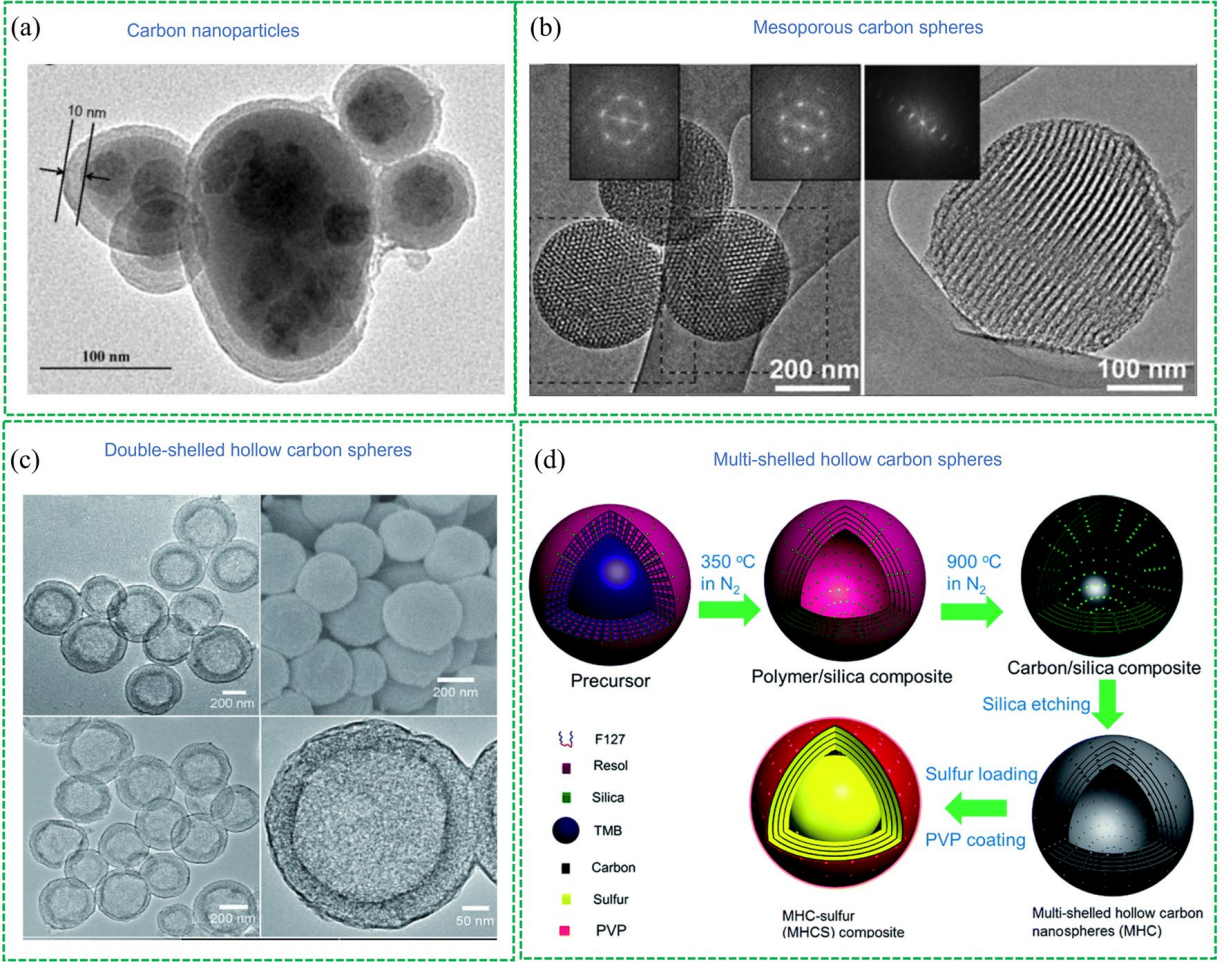
Representative 0 D carbon-based sulfur hosts: Reproduced with permission from Ref. [39]. Copyright 2010 Elsevier;** a** Carbon black. Reproduced with permission from Ref. [40]. Copyright 2012 John Wiley and Sons; **b** Mesoporous carbon sphere. Reproduced with permission from Ref. [46]. Copyright 2012 John Wiley and Sons; **c** Double-shelled hollow carbon spheres. Reproduced with permission from Ref. [47]. Copyright 2014 Royal Society of Chemistry;** d** Multi-shelled hollow carbon spheres

0D materials refer to nanomaterials with all the dimensions within the nanoscale. Carbon black is a representative example and it was proved that loading sulfur on the carbon black can significantly improve the electrochemical performance compared with a pure sulfur cathode (Fig. 4a) [39]. In this system, homogeneous high sulfur loading can be achieved via the chemical depiction method but this sulfur coating shields the electron transfer channel. To mitigate the intrinsic problem of carbon nanoparticles, Nazar’s group developed the spherical ordered mesoporous carbon nanoparticles as sulfur host materials (Fig. 4b) [40]. This material showed high inner pore volumes of 2.3 cm^3^ g^−1^ and high surface areas of 2,445 m^2^ g^–1^ with a bimodal pore size distribution of large and small mesopores of 6 and 3.1 nm. As the sulfur host, this material showed much better performance than nano-sized and bulk carbon, and it was proved that the nanoscale morphology of the mesoporous carbon is beneficial to the preparation of homogeneous C/S composite charge transfer. A porosity tailoring strategy like this was also applied to other nanoscale carbon materials to obtain excellent cathode materials [41, 42]. But the micropores are not friendly to sulfur. The microporous C/S cathode shows a lower discharge plateau and requires a lower discharge voltage (about 1.0 V) to fully release its capacity, compared to large-pores carbon materials requiring > 1.5 V discharge voltage [43]. This is because the diameters of ethylene carbonate (EC) and dimethyl carbonate (DMC) molecular are relatively too large (with theoretically calculated diameters of 0.57 and 0.79 nm) to enter narrow pores, preventing the irreversible reaction between the carbonate and the PS [38].

Encapsulating sulfur in the interior void space can improve the utilization of sulfur and increase the amount of sulfur confined. Hence, hollow porous materials (HPCM) have been paid more attention due to their high specific surface areas, tuneable pores structures, available cavities and controllable morphologies [44]. A classic example can be traced back to the work of Archer’s group in 2011 [45], in which the hollow carbon spheres with a mesoporous shell were developed to obtain cathode material showing high sulfur loading and good electrochemical performance. As a result, the C/S composite with 70 wt% of sulfur exhibited an initial capacity of 1,100 mAh g^–1^ and maintained a reversible capacity of 974 mAh g^–1^ after 100 cycles at 0.5C. It was proved that the special hollow structure can provide fast ion and electron transport and prohibit the shuttle effect via physical adsorption of LiPSs. To maximize the advantages of hollow carbon materials, multi-shelled HPCM has been developed as sulfur host materials. For example, Lou and co-workers [46] reported the double-shelled hollow carbon spheres (Fig. 4c) as sulfur hosts which can effectively encapsulate a high amount of sulfur (64 wt%), prevent the internal polysulfides from diffusing outside of the shell and accommodate volume expansion during cycling. In addition, Wang and co-workers [47] synthesized the multi-shelled hollow carbon (Fig. 4d) achieving an extremely high sulfur loading of 86 wt%. This cathode delivered a high specific capacity of 1,350 mAh g^–1^ at 0.1C and excellent cycling stability with 92% capacity retention after 200 cycles.

###  1D Nanomaterial

**Fig. 5:**
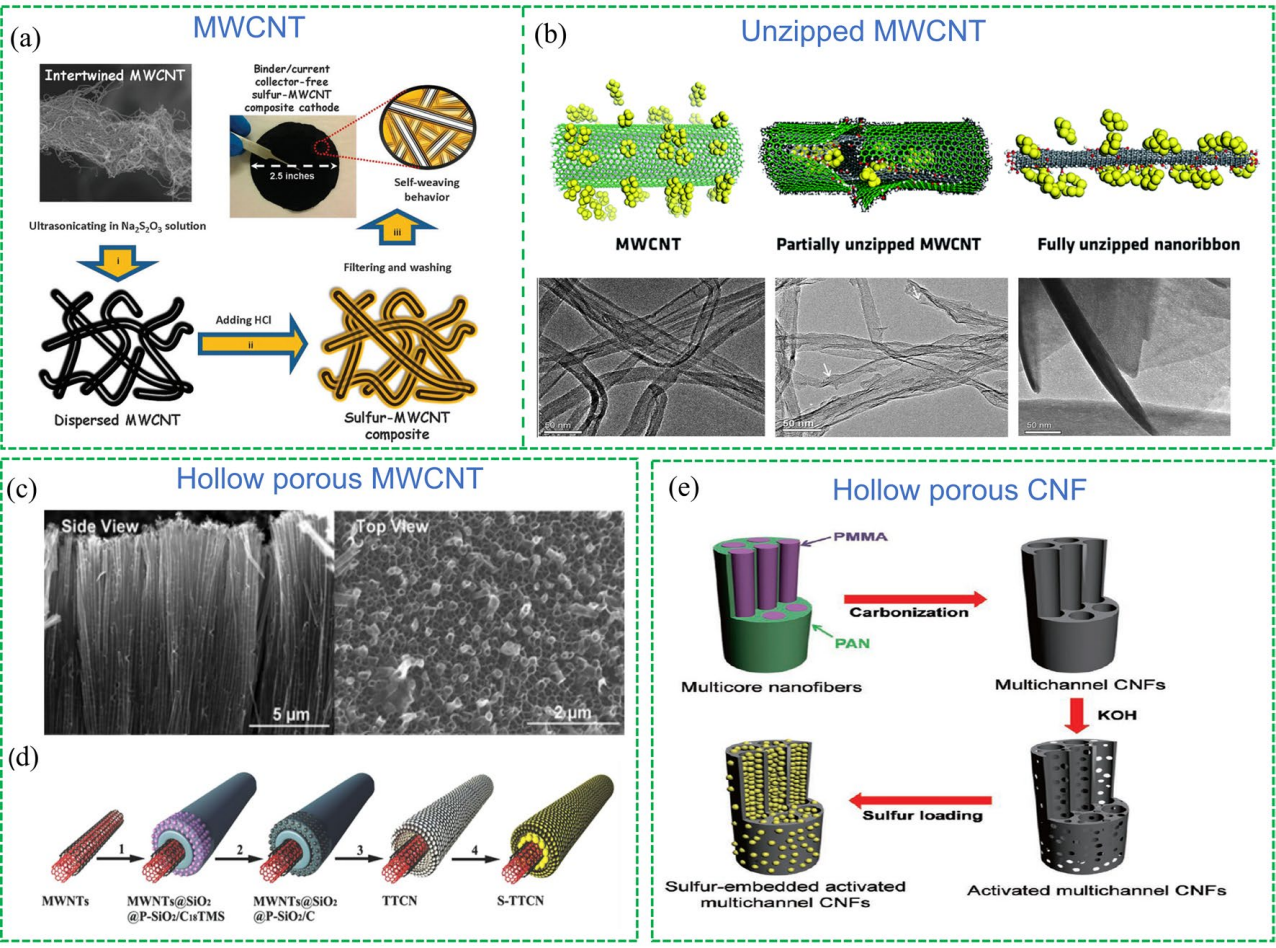
Classic strategies in developing 1 D carbon nanomaterials as sulfur hosts. Reproduced with permission from Ref. [50]. Copyright 2012 Royal Society of Chemistry; **a** MWCNTs. Reproduced with permission from Ref. [51]. Copyright 2016 Royal Society of Chemistry; **b** unzipped MWCNTs. Reproduced with permission from Ref. [52]. Copyright 2011 American Chemical Society; **c** porous disordered carbon nanotubes. Reproduced with permission from Ref. [53]. Copyright 2014 John Wiley and Sons; **d** “tube in tube” MWCNTs. Reproduced with permission from Ref. [54] Copyright 2016 John Wiley and Sons; **e** multichannel CNFs

Carbon nanotubes (CNTs), composed of curved graphene sheets, are typically categorized as single-walled CNTs (SWCNTs) or multi-walled CNTs (MWCNTs) based on the number of graphene layers they contain [48]. SWCNTs exhibit excellent electrical (especially super electrons mobility) and mechanical properties, but the preparation process of SWCNTs is costly [49]. On the other hand, MWCNTs are more economical and do not compromise electrical and mechanical properties, making them an attractive option as sulfur hosts in Li–S batteries. Manthiram et al. [50] adopted in situ sulfur deposition method to load sulfur on MWCNTs to prepare S/C composite (Fig. 5a). Due to the self-weaving nature of the MWCNTs, the prepared S/C composite can be vacuum filtered to form a binder/current collector-free cathode, thereby streamlining the manufacturing process of the electrode and decreasing the whole weight of a cell.

CNTs typically have closed ends, making it difficult to encapsulate the sulfur into the inner channels of CNTs and suppress the diffusion of LiPSs. Similarly, porous or hollow CNTs and carbon nanofibers (CNFs) were developed to effectively confine sulfur. For instance, Park and co-workers [51] reported partially unzipped MWCNTs to increase surface area and pore volume, providing accessible inner channels to confine sulfur and polysulfides with a retained electron conduction pathway (Fig. 5b). As a result, the partially unzipped MWCNTs/S composite exhibited an initial capacity of 708 mAh g^–1^ and retained 570 mAh g^–1^ after 200 cycles at a high current density (5C). Wang et al. [52] prepared the porous disordered carbon nanotubes with open ends as a sulfur host via the template method (Fig. 5c). The sulfur was vaporized and incorporated into graphitized carbon layers and small voids in amorphous carbon that electrolytes cannot directly access. The heat treatment of the sulfur incorporation method could break down the S_8_ molecule to S_6_ and S_2_, and hence the conventional reaction of Li and S_8_ with soluble polysulfide intermediate products could be decreased. This kind of hollow porous MWCNTs normally exhibits relative low conductivity due to the low graphitization. To improve the electrical conductivity without compromising the favourable structural features of hollow nanotubes, a “tube in tube” (TT-CNT) structure with highly graphitized and conductive MWCNTs inside the outer porous tube has been proposed by Zhao et al. [53] As shown in Fig. 5d, the coaxial-structure TT-CNTs were synthesized by a multi-step coating strategy. MWCNTs were firstly coated by solid SiO_2_ and another layer of SiO_2_, of which an organosilicon was then impregnated into the pores to form carbon after calcination. After that, all the silica was removed by NaOH etching to expose the carbon structure. A high S loading (71%) S/TT-CNT composite was obtained and it showed good rate performance which can be attributed to the novel structure: the outer mesoporous carbon tube can host sulfur and prevent the diffusion of polysulfides while the inner MWCNTs facilitate electrons transferring and to booster the electrochemical process.

In addition to carbon nanotubes, carbon nanofibers (CNFs) with intrinsic high conductivity are also attractive in Li–S batteries. For example, Manthiram et al. proposed the activated multichannel CNFs using a single-nozzle co-electrospinning technique (Fig. 5e) [54]. The multichannel CNFs were then activated with KOH to generate micropores on the channel walls. Due to the novel structure, the sulfur loading and sulfur utilization could be enhanced by the mesopores and the micropores could provide trapping sites for anchoring polysulfides. The porous multichannel CNFs could accommodate a high sulfur loading (80%) and exhibited 847 mAh g^–1^ at 5C. This study suggested an effective strategy to solve the problems of low sulfur utilization and rapid capacity fade in Li–S batteries.

### 2D Materials

The most popular 2D carbon material used in Li–S batteries is graphene. Graphene has a large theoretical specific area of 2,630 m^2^ g^–1^ [55] and a high charge carrier mobility (> 2 × 10^5^ cm^2^ V^−1^ s^−1^), making it a promising candidate for Li–S batteries. Chen et al. [55] used the reduced graphene oxide (rGO) as the sulfur host and investigate the effect of the sizes of sulfur particles on the performance. Nanoparticles with average diameters ranging from 150 to 5 nm on the rGO were uniformly dispersed on the rGO, and it was proved that the sulfur nanoparticles showed better electrochemical performance with decreased particle size. As a result, the rGO/S composite loaded with 5 nm sulfur nanoparticles exhibited the specific capacity of 1,672 mAh g^–1^ at 0.1C (theoretical specific capacity) and 1,089 mAh g^–1^ at 4C.

**Fig. 6:**
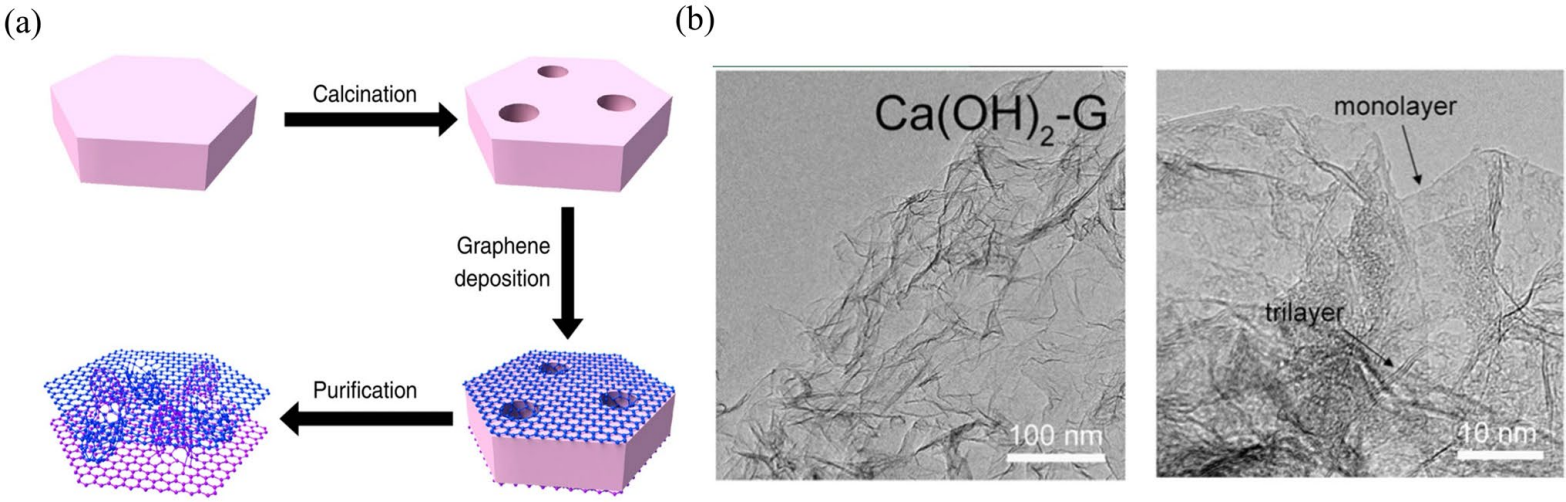
Reproduced with permission from Ref. [56]. Copyright 2014 Springer Nature; **a** Schematic illustration of the synthesis process of DTG. Reproduced with permission from Ref. [57]. Copyright 2015 John Wiley and Sons; **b** TEM images of graphene nanosheets cast onto LDO flakes

In the process of synthesising graphene, the graphene layers are more likely to stack layer by layer due to the huge surface area and strong π–π interactions between graphene layers, and this stacking leads to a smaller surface area and poor energy-storage performance. To prevent the stacking of graphene and improve the confinement capability of sulfur, Zhao et al. [56] proposed a template directed method to prepare unstacked double-layer template graphene (DTG) composed of two graphene layers separated by a large amount of mesosized protuberance. The process is shown in Fig. 6a, the MgAl-layered double hydroxide (LDH) nanoflakes were used as templates to prepare unstacked graphene via chemical vapor deposition. Mesopores are formed in the LDHs during the calcination process so that the LDHs can not only prevent the stacking of graphene layers but also induce the formation of mesopores in graphene. As a result, the surface area of the prepared DTG reached 1,628 m^2^ g^–1^, and the DTG/S cathode showed excellent high-rate performance, achieving high reversible capacities of 1,035 and 735 mAh g^–1^ at a discharge rate of 5C and 10C respectively. Similarly, Zhang et al. [57] used the CaO as a template and a catalyst to enhance the growth of graphene with a hierarchical porous structure using chemical vapor deposition (Fig. 6b). As a sulfur host, it exhibited better cyclic stability and rate performance in Li–S batteries.

### 3D Materials

3D carbon materials offer the opportunity to combine the benefits of materials with different dimensions, and their structures can be tailored to meet diverse requirements. 3D porous carbon materials, in particular, hold great promise as sulfur hosts in Li–S batteries, as they not only retain the advantages of lower-dimensional materials, but also offer additional benefits such as high tap density, rapid charge transport, and a hierarchically porous structure [58, [59].

**Fig. 7:**
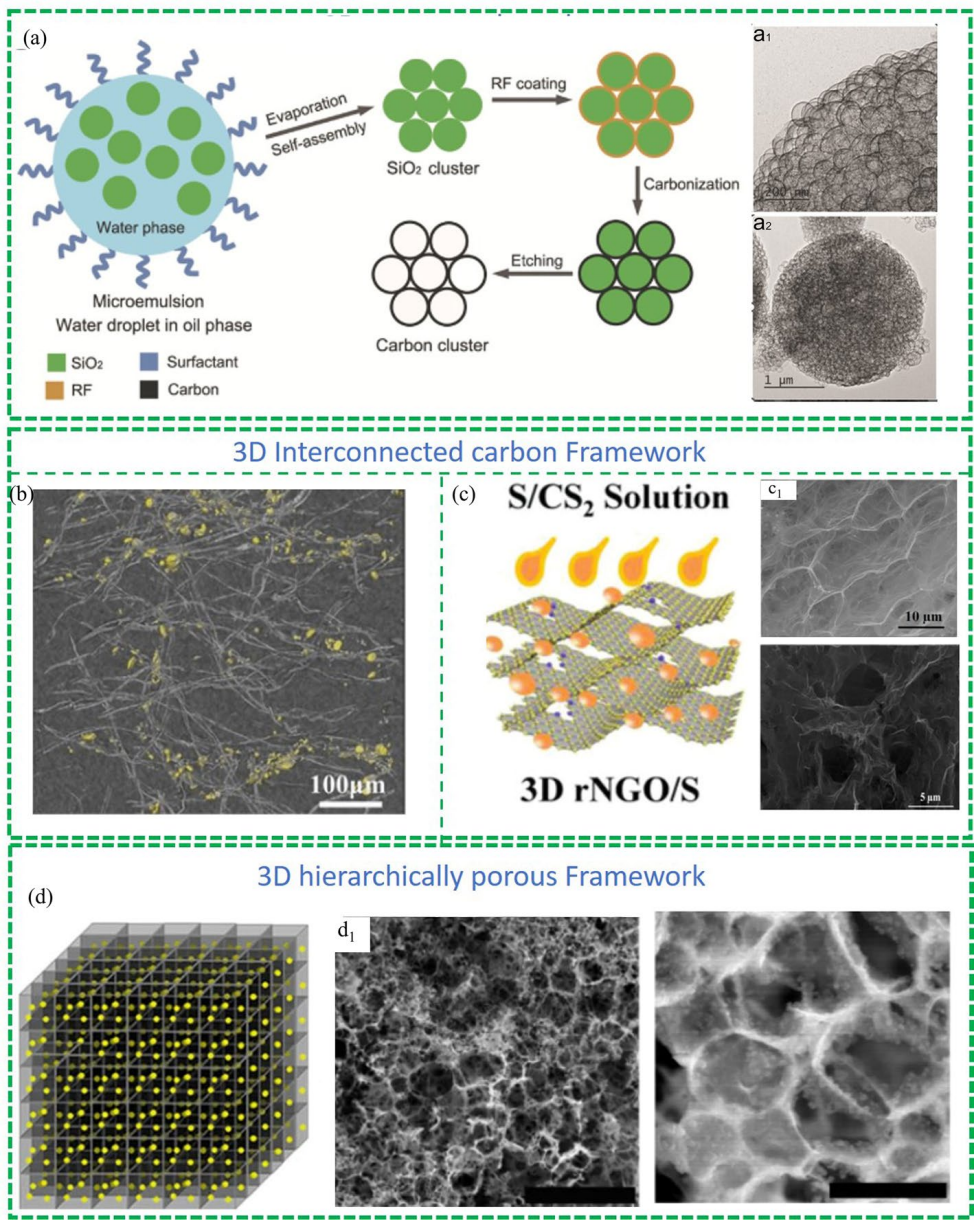
Representative 3D carbon materials as cathodes in Li–S batteries: Reproduced with permission from Ref. [60]. Copyright 2015 John Wiley and Sons; **a** 3D porous carbon spheres cluster. 3D interconnected carbon framework: Reproduced with permission from Ref. [61]. Copyright 2016 John Wiley and Sons; **b** 3D integrated hollow carbon fibre foam. Reproduced with permission from Ref. [62]. Copyright 2019 John Wiley and Sons; **c** 3D porous graphene framework. Reproduced with permission from Ref. [63]. Copyright 2016 Springer Nature; **d** 3D hierarchically porous framework

One way to form a 3D carbon framework is through the interaction of nanocarbon materials. For example, Cui et al. [60] reported a method to prepare the pomegranate-like hierarchically porous carbon sphere (HPC) clusters. As shown in Fig. 7a, the preassembled SiO_2_ spheres were used as the porous structured template and RF as the carbon precursor. Transmission electron microscopy (TEM) images in Fig. 7a (a1 and a2) showed that the average diameter of the primary HPCs was approximately 120 nm, and the thickness of the primary particles was calculated to be 1–2 nm for the inner shell and 4–5 nm for the outer shell. These hierarchically porous carbon clusters displayed a high sulfur loading capacity and improved the suppression of the polysulfide shuttling effect since the sulfur species in the inner cores were less likely to diffuse out. To further improve the cyclic stability of the material, a layer of conductive polymer, poly(3,4-ethylenedioxythiophene) (PEDOT), was coated on the HPCs/S clusters to serve as a protective layer to inhibit polysulfides diffusion. The cell with HPCs/S clusters as cathode delivered a high reversible specific capacity of more than 700 mAh g^–1^ at a high current rate of 3C.

In another study, Fang et al. synthesised a 3D integrated hollow carbon fibre foam (HCFF) and obtain electrodes with ultrahigh sulfur loading of 21.1 mg cm^−2^. (Fig. 7b) [61]. Due to the high electrolyte absorbability and multiple conductive channels which contribute to the accelerated redox kinetics of the sulfur and localised polysulfides, the HCFF-S electrodes exhibited excellent cycling stability with a high capacity retention of 70% over 150 cycles at under a high sulfur loading with 21.1 mg cm^−2^. Similarly, Duan et al. [62] reported a kind of 3D porous graphene framework to serve as a sulfur host (as shown in Fig. 7c). This porous conductive matrix can not only facilitate the fast migration of electrons and ions but also accommodate high volume sulfur. The cathode with an extremely high S loading of 90% was obtained, delivering a specific capacity of 1,186 mAh g^–1^ after 200 cycles at 0.1C and 578 mAh g^–1^ after 1,000 cycles at 1.0C. The template method is also a widely-used method to prepare 3D carbon materials. For instance, Li et al. reported an in-site template method to prepare 3D porous carbon composites containing sulfur nanoparticles (3D PGC/S) (Fig. 7d) [63]. The 3D PGC/S electrodes, prepared through the nanoscale dispersion of sulfur particles and the formation of covalent bonds between sulfur and the carbon network, exhibited exceptional electrochemical performance characterized by high sulfur utilization. Specifically, the electrodes demonstrated capacity of 1,242 and 1,115 mAh g^–1^ at 1C and 2C, respectively, while experiencing only a minor capacity decay of 0.039% per cycle over a span of more than 1,000 cycles at 2C.

Various carbon materials have been designed with different structures, and these designs have proven to be effective in enhancing the electrochemical performance of these materials as hosts for sulfur. Some representative carbon-based sulfur hosts with various structures have been compared in Table 1. The fundamental approach is centered on fabricating highly conductive carbon matrices capable of proficiently enclosing additional sulfur and affording augmented physical adsorption channels for polysulfides, including porous, hollow, and core/shell structures. Although the noteworthy hindrance of LiPSs diffusion attributable to the physical confinement and adsorption of carbon matrices, complete inhibition of the shuttle effect is impeded by the feeble interaction between the nonpolar carbon matrix and polar LiPSs. To surmount this limitation, chemical modification techniques have been advanced based on these structure designs (Table 2).

**Table 1:** Comparison of carbon-based sulfur hosts with different structures

Sulfur hosts		Sulfur loading (mg cm^−2^)	Capacity (mAh g^–1^) (low rate)	Capacity (mAh g^–1^) (high rate)	Capacity retention(cycles and rates)	Refs.
0 D	double-shelled hollow carbon spheres	< 2	1020 (0.1C)	350 (1C)	74% (100 cycles at 1C)	[46]
	Porous carbon spheres	2.07	1015 (0.2C)	875 (1C)	90% (100 cycles at 1C)	[41]
	Multi-shelled hollow carbon nanospheres	/	1350 (0.1C)	1003 (1C)	84% (200 cycles at 1C)	[47]
	porous hollow CNTs@CNFs	/	1313 (0.2C)	572 (5C)	80% (100 cycles at 1C)	[64]
1D	Partially unzipped carbon nanotubes	/	1301.8 (0.2C)	688.5 (5C)	80% (200 cycles at 5C)	[51]
	Multichannel Carbon Nanofiber	2.2	1351 (0.2C)	847 (5C)	79% (300 cycles at 0.2C)	[54]
	Double-layer graphene	1.1	1200 (0.5C)	1034 (5C)	80.5% (200 cycles at 5C)	[56]
2D	Hierarchical Porous Graphene	2.0	1053 (0.5C)	783 (5C)	84.5% (150 cycles at 0.5C)	[57]
	Hierarchical Porous Carbon Rods	1.5	972 (0.5C)	646 (5C)	72% (300 cycles at 1C)	[59]
3D	Pomegranate-Like Carbon Cluster	2	1020 (0.5C)	733 (3C)	68.62% (300 cycles at 0.5C)	[60]
	Three-dimensional porous carbon	2.36	1382 (0.5C)	1115 (2C)	83% (200 cycles at 2C)	[63]

**Table 2:** Comparison of different sulfur hosts developed by chemical modification startagies

Strategies	Sulfur hosts	Sulfur loading (mg cm^−2^)	Capacity (mAh g^–1^) (low rate)	Capacity (mAh g^–1^) (high rate)	Capacity retention (cycles and rates)	Refs.
Functional groups and polymers	Core–shell polymer-coated C/S	1.5	1140 (0.1C)	740 (1C)	85.1% (600 cycles at 0.6C)	[69]
	PANi/S/CNF	2	1074 (0.1C)	570 (1C)	76% (300 cycles at 1C)	[70]
Heteroatom doping	3D N/S co-doped graphene	4.6	1200 (0.2C)	430 (2C)	72.4% (200 cycles at 0.5C)	[75]
	P, N co-doped Double-Shelled Carbon Nanospheres	5.8	1326 (0.1C)	814 (1C)	85.5% (500 cycles at 1C)	[76]
Metal compounds	Porous VN nanoribbon/graphene	3	1,471 (0.2C)	1148 (1C)	85% (100 cycles at 0.2C)	[83]
	Co/Co_3_O_4_-NHC	4	957.1 (0.2C)	538.2 (2C)	87.8% (250 cycles at 1C)	[87]
Lewis-acid	ZDC@ZIF-8	6.9	1361 (0.2C)	751 (2C)	61% (300 cycles at 1C)	[92]
	3D porous MXene/rGO	6	1270 (0.2C)	977 (1C)	65% (500 cycles at 1C)	[94]
Heterostructure	TiO_2_–TiN heterostructures/C	3.1	1300 (0.1C)	900 (1C)	92% (300 cycles at 0.3C)	[99]
	TiO_2_–Ni_3_S_2_/rGO	3.9	1270 (0.1C)	797 (1C)	65% (500 cycles at 0.3C)	[105]
Facet Engineering	Active SnO_2_/rGO	1.3	1074 (0.2C)	701 (2C)	73% (500 cycles at 0.5C)	[113]
Alloying	Ni–Pt alloy/ graphene	4	783.3 (0.1C)	664.9 (1C)	75% (1000 cycles at 1C)	[115]
	CoFe alloy/ mesoporous carbon sphere	4.0	1099.2 (0.2C)	671.4 (1C)	70% (500 cycles at 2C)	[114]
Single atom tailoring	single-atom Ti/ carbon foams	4	1151 (0.2C)	698 (1C)	86.5% (300 cycles at 0.5C)	[120]
	single-atom Fe/ g-C_3_N_4_	2.3	1379 (0.1C)	704 (5C)	90% (200 cycles at 0.2C)	[121]

## Chemical Modification

### Chemical Anchoring of Sulfur Species

To further enhance the anchoring ability toward LiPSs, chemical adsorption via chemical bonds with atoms or molecules has been paid more attention. Chemical interaction between sulfur hosts and LiPSs displays more robust absorbability than physical confinement, thereby impeding the escape of polysulfides from conductive matrices. Based on the modes of chemical bonds between sulfur hosts and polysulfides, chemical interactions can be classified into two groups: polar-polar bonds and Lewis acid–base bonds. To attain optimal sulfur hosts, more chemically active sites need to be introduced into the materials to generate chemical interactions with polysulfides. Therefore, structurally optimized carbon materials are the most favorable host materials since they can not only offer more areas to localize chemical active sites but also leverage their other merits such as high conductivity networks, physical confinement of polysulfides, and high sulfur loading.

#### Polar-Polar Bonds

Because of the polarity of polysulfides, many methods that can introduce polar molecules or atoms into porous carbon materials have been developed, including functional groups and polymers, heteroatom doping, carbon nitride (g-C_3_N_4_), boron nitride (BN) and metal compounds.

**Fig. 8:**
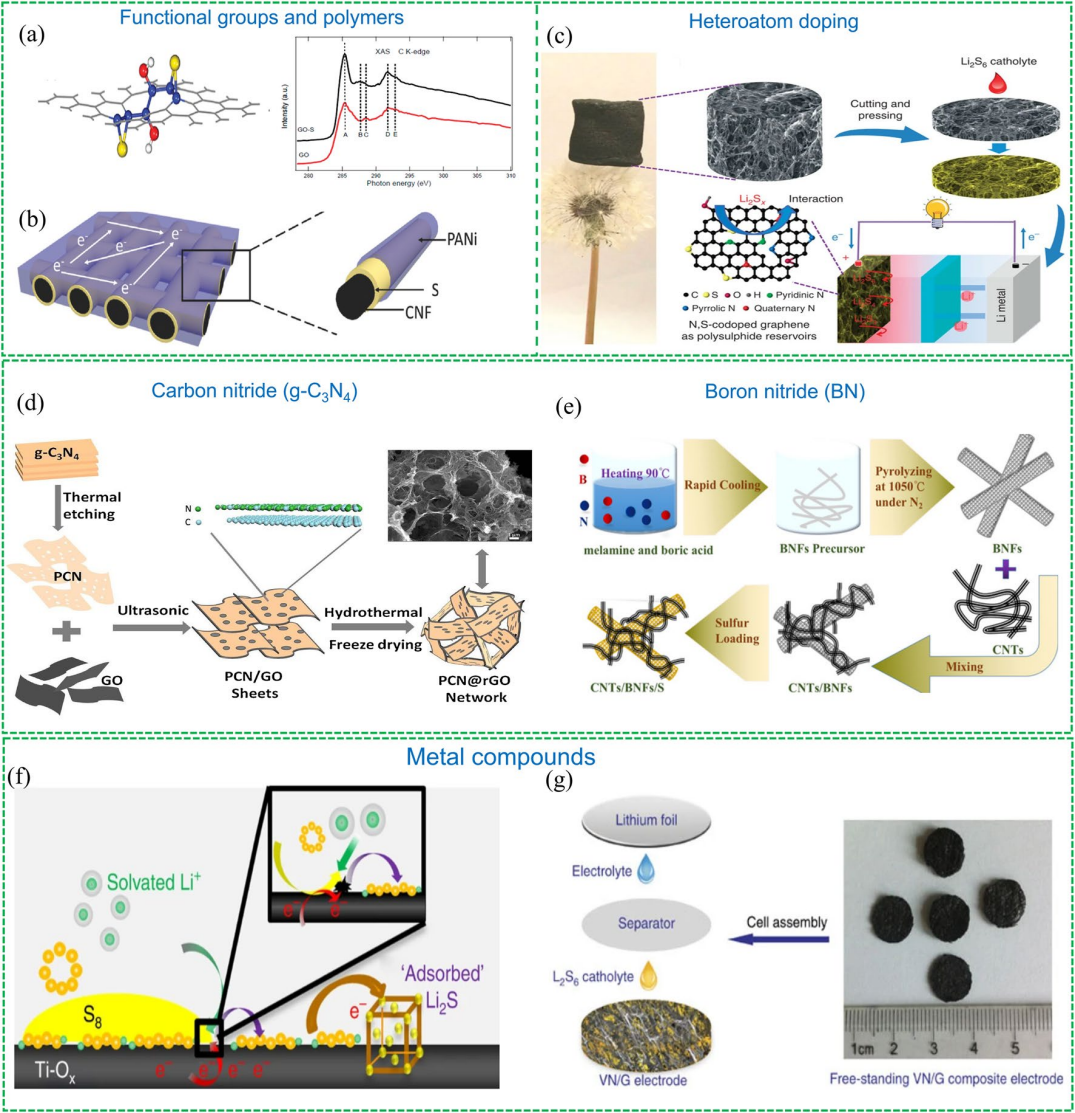
Representative examples of cathode materials modified by different chemical anchoring strategies. Reproduced with permission from Ref. [66]. Copyright 2011 American Chemical Society; **a** Schematic illustration of GO with adsorbed sulfur species, C K-edge XAS spectra of GO and GO-S. Reproduced with permission from Ref. [70]. Copyright 2018 John Wiley and Sons; **b** Schematic of the configuration of CNF/S/PANI composite electrode. Reproduced with permission from Ref. [75]. Copyright 2015 Springer Nature; **c** Illustration of the 3D N, S co-doped graphene sponge electrodes and its working principle as a sulfur host. Reproduced with permission from Ref. [79]. Copyright 2017 Elsevier; **d** Preparation procedures of the 3D PCN@rGO network composite. Reproduced with permission from Ref. [81]. Copyright 2020 John Wiley and Sons; **e** Schematic illustration of the preparation method of CNTs/BNFs/S. Reproduced with permission from Ref. [82] Copyright 2014 Springer Nature; **f** Illustration of the interaction between Ti_4_O_7_ and LiPSs. Reproduced with permission from Ref. [83]. Copyright 2017 Springer Nature; **g** Preparation process of the VN/G composite

*Functional groups and polymers:* Introducing some functional groups that can interact with sulfur species in carbon substrates is an effective way to improve the cyclic stability of carbon-based materials as sulfur hosts [65]. Graphene oxides (GO) has been studied as sulfur host to inhibit soluble LiPSs due to the abundant oxygenated functional groups. Ji et al. prepared S/GO composite by depositing nano sulfur on GO sheets and subsequently heating it in an argon environment at 155 °C [66]. The DFT calculation was conducted and it indicated both epoxy and hydroxyl groups can bond with S species (Fig. 8a). The evidence of a chemical bond between S species and GO was analysed by soft X-ray absorption spectroscopy (XAS) measurement. Notably, this approach is facing two critical challenges: (1) the introduction of oxygenated functional groups can sacrifice the electrical conductivity of carbon substrates, (2) the groups can react with Li and electrolyte, resulting in the loss of active sites and accumulation of insulating layers on the electrode surface.

To prevent the diffusion of lithium polysulfides (LiPSs), carbon materials have been grafted with amphiphilic polymers that have plenty of anchoring groups. An initial study found that incorporating polyvinylpyrrolidone (PVP) onto the surface of hollow carbon nanotubes (CNTs) is a useful method to sequester polysulfides [67]. Later, polymers with various functional groups, such as nitriles, amines, esters, thiophenes, and quinonoid-imine, have been combined with carbon materials to improve LiPSs adsorption [68]. Amines groups have been widely studied in carbon substrates like CNTs, graphene oxide (GO), and hollow carbon spheres [69]. For instance, Zhou et al. fabricated a double-layered core–shell polymer-coated C/S composite [69]. They impregnated sulfur into the hollow carbon spheres under heat treatment, and then coated the surface of the carbon spheres with polydopamine (PDA) via in-situ polymerization to form a double-layered core–shell structure. The hollow carbon shells controlled the internal sulfur size and served as a conductive layer to promote electrons transfer, while the coating polymer constrained sulfur and polysulfides due to strong chemical adsorption. However, introducing insulating polymer layers to carbon materials poses similar problems to functional groups. Therefore, conductive polymers with polar bonds have also been studied. Zhu et al. developed a simple and effective method to produce a PANi/S/CNF cathode by in-situ polymerizing PANi on 3D carbon fiber networks, as shown in Fig. 8b [70]. The interconnected CNF/PANi network structure facilitated electron transfer, while the abundant nitrogen heteroatoms provided strong LiPSs adsorption. A binder-free cathode was achieved, exhibiting high S utilization and a low capacity decay of 0.08% per cycle for over 300 cycles.

*Heteroatom doping:* The modification of surface electronic structures of carbon matrices to generate polar sites via heteroatom doping has been found to be an effective method for inhibiting polysulfide diffusion. Song et al. proposed that N-doped carbon materials can effectively immobilize LiPSs in 2013 [71]. The experimental characterisation like X-ray absorption near edge structure spectroscopy and density functional theory (DFT) simulation successfully confirmed this concept. To further understand the mechanisms of anchoring LiPSs by N-doped carbon, various studies on surface chemistry have been conducted. There are three different N species: pyrrolic N, pyridinic N and graphitic N, and it was proved that only the pyridinic N can anchor LiPSs with strong binding because of the enhanced attraction between Li ions in LiPSs and the pyridinic N dopant. The chemical interaction between the sulfur species and pyridinic N dopant was also experimentally confirmed by detecting carbon-bonded thiophene-like sulfur and highly oxidized sulfur species SO_x_ [72].

In addition to the widely-studied N doping, other elements including O, P, S, B, F, Cl and Se have been investigated as well. Apart from O dopants, these single-atom dopants are not satisfactory due to their weak binding with sulfur species [73]. But dual doping of N and other elements like B, P, S and O was proved to be an effective way to enhance the interaction between LiPSs and carbon matrixes [74]. For instance, Zhou et al., presented a 3D N, S co-doped graphene sponge electrode which can provide enough space for a high sulfur loading, facilitate electrons and ions transfer and strongly anchor polysulfide [75]. As shown in Fig. 8c, the sponge is light and can stand on the top of a dandelion without deforming. It can be pressed and cut into slices, and directly used as a host for sulfur species without metal current collections, conductive additives and binders, showing strong chemical adsorption of polysulphide. Similarly, a versatile strategy to prepare double-shelled N, P co-doped carbon spheres (NPDSCS) has been reported by Wang and co-workers [76]. As a sulfur host, the NPDSCS exhibited enhanced affinity and trapping ability towards LiPSs, thereby showing excellent cycling performance. Although heteroatom doping is an effective approach to improve the anchoring capability of carbon materials toward polysulfides via chemical bonding, it is challenging to increase the concentration of dopants, for instance, the N-doping concentration is usually less than 15% in doped carbon materials [77].

*Carbon nitride (g-C*_*3*_*N*_*4*_*) and Boron nitride (BN):* Graphitic carbon nitride (g-C_3_N_4_), a highly ordered polymeric material consisting of tri-s-triazine units connected to planar amino groups, was reported as a promising functional sulfur host due to its simple fabrication process and high nitrogen content that can provide LiPSs binding sites [77]. The limited surface area and relatively low electrical conductivity are the unsatisfactory aspects of g-C_3_N_4._ Therefore, a variety of methods have been developed to improve the electrical conductivity and specific surface area of g-C_3_N_4_-based material. For instance, Kim et al. prepared a hierarchical tubular C_3_N_4_ based nanomaterial to increase the surface area and provide more active sites [78]. In this work, Fe_3_O_4_ nanospheres were also decorated on the surface of the C_3_N_4_ nanotube to further improve the adsorption of polysulfides and enhance electron transfer. The composite was used as an interlayer of Li–S batteries and showed significant improvements in cyclic performance in comparison with cells without this layer. Combining carbon materials with g-C_3_N_4_ is an effective way to make full use of the virtues of g-C_3_N_4_. As shown in Fig. 8d, Kuang’s group reported a strategy to prepare a 3D light-weight and porous C_3_N_4_ nanosheets@reduced graphene oxide (PCN@rGO) composite [79]. The ultrathin and porous g-C_3_N_4_ (PCN) sheets were firstly prepared to improve the specific surface area, and they were mixed with GO to form a lay-by-lay composite via self-assembly. The structure of this PCN@rGO would form a 3D network via a hydrothermal approach followed by freeze-drying. In this 3D network, the PCN layer can offer substantial chemical anchoring sites while the rGO layer facilitates fast electron transfer, thereby achieving high sulfur utilization.

Similarly, BN nanosheets have been investigated to serve as sulfur hosts and interlayers of Li–S batteries due to their abundant absorption sites. Yi et al. developed a few-layer BN with engineered vacancies (v-BN) as a cathode matrix for Li–S batteries [80]. The vacancies in the BN nanosheets can not only enhance the immobilization of LiPSs, but also promote the Li-ions diffusion in cathode electrodes. Li et al. developed a method to intertwin porous boron nitride fibers (BNFs) and multi-walled carbon nanotubes (CNTs), which enables strong chemical interaction with LiPSs, high sulfur loading and excellent conductivity (Fig. 8e) [81]. The interconnected structure enables good electrolyte uptake and can effectively localize the soluble LiPSs.

*Metal compounds:* Compared to carbon-based materials, metal-based materials that are rich in oxygen, nitrogen, and sulfur atoms provide strong immobilization of lithium polysulfides (LiPSs) and have therefore been explored as potential hosts for sulfur in lithium-sulfur (Li–S) batteries. Among them, metal oxides have been widely reported to anchor LiPSs by polar-polar interaction, including metal-S or O-Li bonds. Liang et al. firstly developed the hydrogen-reduced TiO_2_ to mitigate polysulfide diffusion and thus improve capacity retention [84] Subsequently, Magneli-phase Ti_4_O_7_ which contains polar O-Ti–O units exhibiting a strong affinity for LiPSs has been reported by Nazar’s group (Fig. 8f) [82]. Visual adsorption investigations with X-ray photoelectron spectroscopic (XPS) and X-ray absorption nearedge structure (XANES) studies confirmed the strong metal oxide-polysulfide chemical interactions. Due to the strong interaction between Ti_4_O_7_ and LiPSs, the LiPSs can be adsorbed on the surface of the cathode and reduced to Li_2_S via surface-mediated reduction at the interface, which is illustrated in Fig. 8f. This work opened the path to investigate functional material with metal compounds that offer strong chemical absorption to migrate the “shuttle effect”. Other metal oxides including MoO_2_ [85], Co_3_O_4_ [86], Fe_3_O_4_ [87] and V_2_O_5_ [88] were also developed or decorated on carbon substrates to serve as cathode materials. Metal nitrides have been paid much attention to due to their excellent electrical conductivity and chemical binding interactions with LiPSs. Vanadium nitride (VN) showing a high electrical conductivity (1.17 × 10^6^ S m^−1^ at room temperature) and strong absorption for polysulfides has been regarded as a promising cathode material for Li–S batteries. Sun et al. developed a 3D highly conductive porous VN/G composite combining the advantages of graphene and VN (Fig. 8g) [83]. The Li_2_S_6_ catholyte was added to the free-standing VN/G plates to prepare the cathode electrode without using carbon black and binder. The 3D network structure of the graphene facilitates the transportation of electrons and lithium ions while the VN strongly anchors the polysulfide. As a result, the VN/G cathode exhibited a high specific capacity of 1,460 mAh g^−1^ at 0.2C and a high rate performance of 960 mAh g^−1^ at 2C.

Other metal nitrides like titanium nitride (TiN) [89], cobalt nitride (Co_4_N) [90] and tungsten nitride (WN) [90], have been investigated. Furthermore, many other metal compounds such as sulfide and carbides with high polarity have been widely studied, some of them can not only offer strong anchoring for polysulfides and exhibit excellent catalytic activity, which will be discussed in the following catalytic cathode section.

#### Lewis-acid

**Fig. 9:**
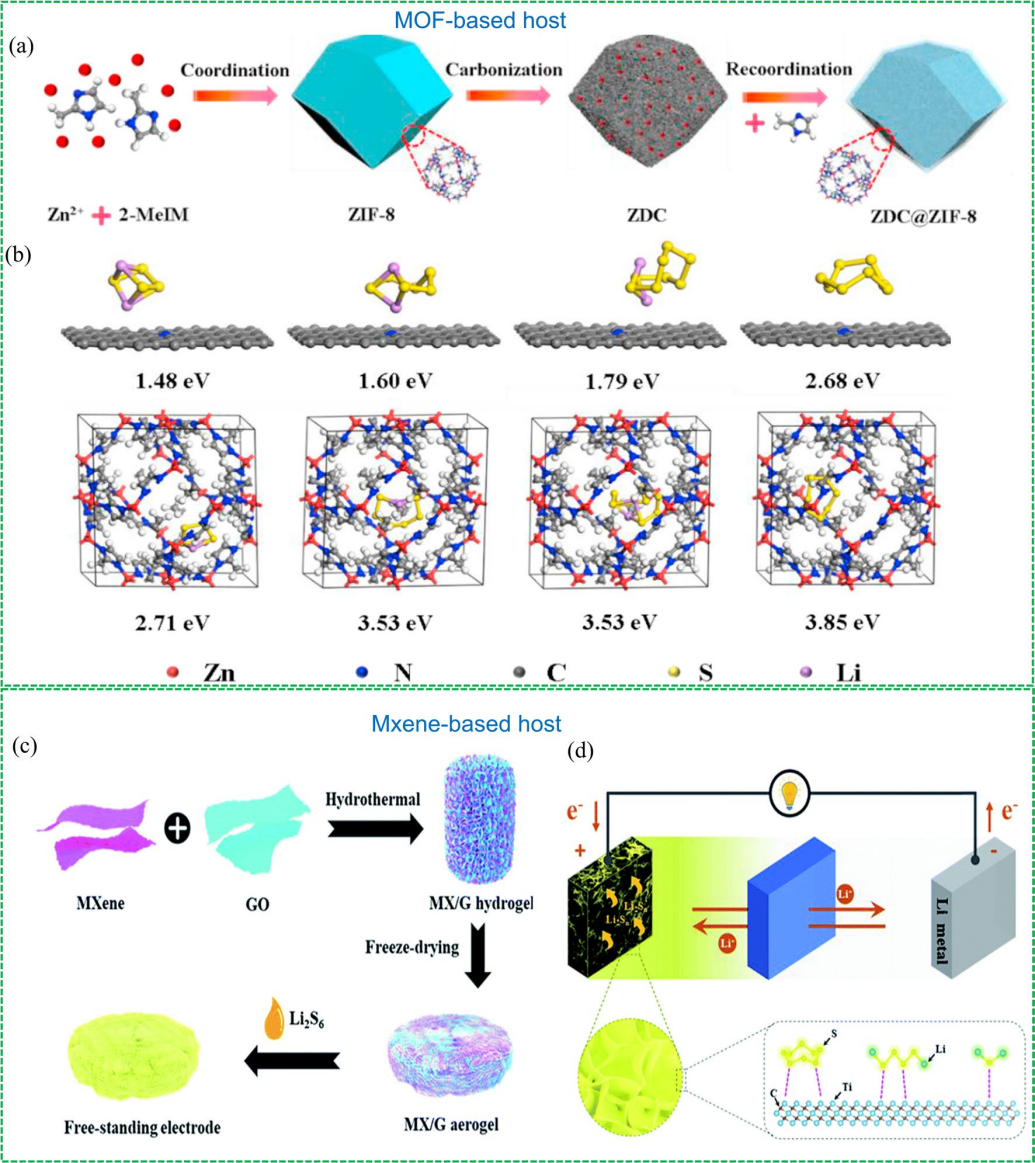
Chemical anchoring strategies and representative examples. MOF-based host: Reproduced with permission from Ref. [92]. Copyright 2019 Elsevier; **a** Illustration of the interaction between Ti_4_O_7_ and **b** the optimized configuration of the binding energy between LiPSs and ZDC@ ZIF-8. Mxene-based host: Reproduced with permission from Ref. [94]. Copyright 2019 Royal Society of Chemistry; **c** Strategy for preparing the MX/G aerogel and **d** schematic of the function of the electrode in a Li–S battery

The polysulfide can be regarded as a Lewis base since the polysulfide anions have occupied orbital with lone electron pairs. Therefore, the chemical species with the property of a Lewis acid can accept lone pair of electrons from polysulfide, thereby anchoring polysulfides by Lewis acid–base interaction. Metal ions in metal–organic framework (MOF) and Mxene are two representative Lewis-acid sites and have been proved to be able to effectively anchor LiPSs via Lewis acid–base interaction [91]. Introducing these materials into carbon materials can give full play to their capabilities. For instance, Pan et al. reported a simple method to prepare a zeolitic imidazolate framework-8 (ZIF-8) derived polyhedral carbon matrix (ZDC) and coat a new ZIF-8 layer on the surface to obtain a composite (ZDC@ZIF-8) (Fig. 9a) [92]. As a sulfur host, the porous structure of ZDC inherited from ZIF-8 and self-doped N atoms offer both physical and chemical interaction with LiPSs, while the metal atoms in ZIF-8 coating prevent the diffusion of LiPSs via Lewis acid–base interaction. The high absorption ability of the ZDC@ZIF-8 was further proved by density functional theory (DFT) calculation. As shown in Fig. 9b, the binding energies of the pure ZDC with Li_2_S_4_, Li_2_S_6_, Li_2_S_8_ and S_8_ are 1.48, 1.60, 1.79 and 2.68 eV, which are lower than those of the coating ZIF-8 layer (2.71, 3.53, 5.53 and 3.85 eV), indicating the ZIF-8 can effectively confine polysulfide in this core–shell structure to alleviate the “shuttle effect. As a result, the ZDC@ZIF-8/S cathode with 74 w% sulfur delivered a high specific capacity of 1,118 mAh g^−1^ with good cycling stability over 300 cycles at 1C.

Similarly, the Lewis-acid Mxene has been also introduced into carbon materials to prepare composites with different structures [93]. Wang’s group rationally designed a unique 3D porous Ti_3_C_2_T_*x*_ MXene/rGO (MX/G) aerogel and applied it to prepare a free-standing electrode to increase the sulfur content and mitigate the “shuttle effect” (Fig. 9c) [94]. In this method, the Mxene layers interact with GO sheets layer by layer via self-assembly, and then they form a 3D interconnected porous aerogel structure after hydrothermal treatment. The M/G hybrid aerogel showed a 3D interconnected porous structure with Lewis-acid surfaces, thereby offering high electrical conductivity and possessing strong LiPSs anchoring abilities (Fig. 9d). As a result, the MX/G-S electrode delivered excellent rate performance and cyclical stability with a high capacity of 1,270 mA h g^−1^ at 0.1C and a low capacity decay rate of 0.07% per cycle.

### Catalyzing the Conversion of Sulfur Species

Efficient redox reaction kinetics are crucial for mitigating capacity decay and restricting the shuttle effect in Li–S batteries due to the sluggish conversion between S8 and Li_2_S_2_/Li_2_S, which leads to the irreversible loss of active materials and an increase in redox voltage polarization. In recent years, a variety of methods have been developed to prepare electrocatalysts and optimize their design in cathodes. An ideal electrocatalysis reaction process for the conversion of S species should satisfy three key requirements: (i) abundant triple-phase interfaces (electrocatalyst, electrolyte, and conductive substrate) to enhance redox reaction efficiency, (ii) strong capture and catalytic activity towards polysulfides to accelerate PS conversion, and (iii) highly efficient dissociation for Li_2_S_2_/Li_2_S to prevent dead sulfur formation [95]. To achieve highly efficient electrocatalytic conversion of S species, it is necessary to optimize both the design of conductive substrates and the catalyst. This can be achieved through various modulation strategies, including exposing active sites and altering the electronic structure of the electrocatalyst. The following sections summarize some significant modulation strategies for optimizing catalysts.

#### Heterostructure Engineering

Heterostructure engineering is a surface tuning method that has proven to be effective in enhancing the catalytic reaction activity in electrocatalytic systems. By rationalizing the structure and combining the merits of each component, heterostructure engineering can achieve synergistic performance. Recent studies have shown that various materials, including metals, metal oxides, metal sulfides, metal carbides, and metal nitrides, exhibit catalytic properties for the conversion of LiPSs [96–98]. An ideal S host with catalytic sites should possess excellent electrical and ionic conductivity, high adsorption capacity, and strong catalytic activity for LiPSs. However, independent catalysts are less likely to meet all of these requirements. Therefore, materials with heterostructures have been developed to overcome the limitations of pure catalysts.

**Fig. 10:**
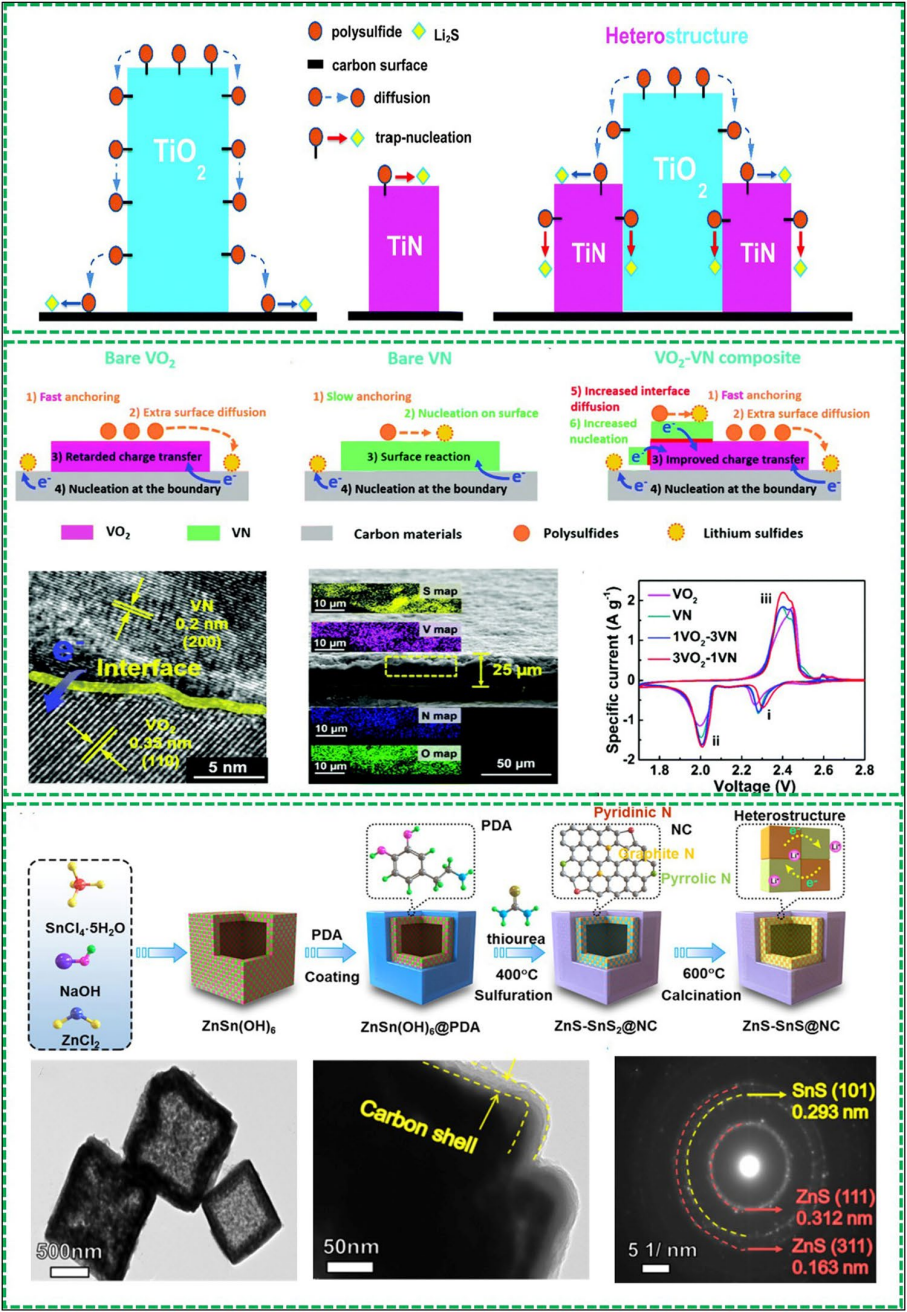
Reproduced with permission from Ref**.** [99]**.** Copyright 2017 Royal Society of Chemistry; **a** Schematic of LiPS conversion processes on TiN, TiO_2_ and the TiO_2_–TiN heterostructure surface. Reproduced with permission from Ref. [100]. Copyright 2018 Royal Society of Chemistry; **b** Schematics of LiPS anchoring-diffusion–conversion processes on VO_2_, VN and VO_2_–VN binary host surfaces. **c** HRTEM images of VO_2_–VN heterostructure. **d** Cross-sectional SEM image of the S@VO_2_–VN/G material and the corresponding element maps. **e** CV curves of different samples. Reproduced with permission from Ref. [101]. Copyright 2021 American Chemical Society. **f** Schematic illustration of the fabrication procedure of ZnS-SnS@NC. **g, h** TEM images of ZnS-SnS@NC. **i** SAED patterns of ZnS-SnS@NC

*Tuning electronic structure:* The catalytic performance of many metal compound catalysts is hindered by their low electrical conductivity, which hinders electron transfer for redox reactions. To address this issue, heterostructures have been introduced to tune the electronic structure of these metal compounds. The TiO_2_-TiN heterostructure is a notable example, as illustrated in Fig. 10a [99]. While TiO_2_ exhibits strong adsorption for polysulfides, its low electrical conductivity inhibits the full involvement of immobilized LiPSs in redox reactions. To enhance conversion, metal compounds are commonly composited with carbon materials, but this approach often results in slow reaction kinetics and impeded adsorption/catalysis due to the accumulation of insoluble Li_2_S on the carbon surface. To address these limitations, TiN, which provides both anchoring sites and fast electron transfer for redox reactions, has been combined with TiO_2_ to form a heterostructure. In this approach, the TiO-TiN heterostructure is loaded onto graphene, where LiPSs are strongly adsorbed on TiO_2_ and then diffuse to TiN, promoting LiPSs nucleation and rapid conversion into insoluble products. Another example of heterostructure engineering is presented by Liu's group, who developed a VO_2_-VN binary sulfur host that combines anchoring and catalytic ability (VO_2_) with good electrical conductivity (VN) to create a high-performance cathode for Li–S batteries [100]. The schematics of LiPSs anchoring-diffusion-conversion process are shown in Fig. 10b. In this design, VO_2_ exhibits anchoring and catalytic ability for LiPSs, but its low electrical conductivity hinders LiPS conversion on the surface. In contrast, VN shows high conversion activity due to its polar and conducting nature, but the chemical bonding of LiPSs on its surface is weaker than on the VO_2_. The VO_2_-VN binary material overcomes these limitations by facilitating electron and S species transfer via excellent interfacial contact between VO_2_ and VN, achieved by the in-situ construction route. The VO_2_-VN binary structure was characterized using exhaustive TEM, and the spatial distribution of elements in the prepared cathode electrode was characterized by elemental mapping in a cross-section SEM view, as shown in Fig. 10c, d. The enhanced redox reaction kinetics for LiPSs were confirmed by CV curves (Fig. 10e), where the reduction peaks shifted to a higher potential and the oxidation peaks shifted to a lower potential for the S@ VO_2_-VN/G cathodes. A recent study proposed a ZnS-SnS heterojunction, which incorporates the merits of strong absorbability and high electrical conductivity of SnS with the efficient catalytic activity of ZnS [101]. The process is shown in Fig. 10f, ZnSn(OH) precursors, which were fabricated by a coprecipitation reaction between Zn^2+^, Sn^4+^, and OH^–^, are utilized as a template and coated with a polydopamine (PDA) shell to form ZnSn(OH)_6_@PDA. After that, the ZnSn(OH)_6_@PDA was converted into ZnS-SnS_2_ heterostructures packaged with a N-doped carbon layer (ZnS-SnS_2_@NC) via in-situ sulfurizations with an excess amount of thiourea at 400 °C under argon atmosphere. The core–shell structural ZnS-SnS@NC was obtained after the calcination at 600 °C. The structure of ZnS-SnS@NC was characterized by SEM and TEM, the cubic morphology with a 20 nm thick carbon outer shell and ZnS-SnS inner shell is shown in Fig. 10g–i. Similarly, some other heterostructures have been developed including ZnS-FeS [102], Co_3_S_4_/MnS [103], and layered double hydroxide (LDH)–Co_9_S_8_ [104].

*Bidirectional electrocatalysts:* The sulfur reduction reaction (SRR) is hindered by low conversion efficiency and detrimental disproportionation reactions of lithium polysulfides (LiPSs), which limits sulfur utilization. On the other hand, the sulfur evolution reaction (SER) faces challenges due to the high activation energy required to dissociate Li_2_S, leading to incomplete sulfur redox kinetics and the presence of "dead" sulfur. In addition, the catalytic activity of heterogeneous catalysts is reliant on surface reactions, and insulating discharge products (Li_2_S/Li_2_S_2_) can cover the limited active sites, prematurely terminating the sulfur redox reaction. Therefore, the development of bidirectional electrocatalysts capable of catalyzing both SRR and SER is crucial to increase sulfur utilization and achieve stable Li–S batteries.

**Fig. 11:**
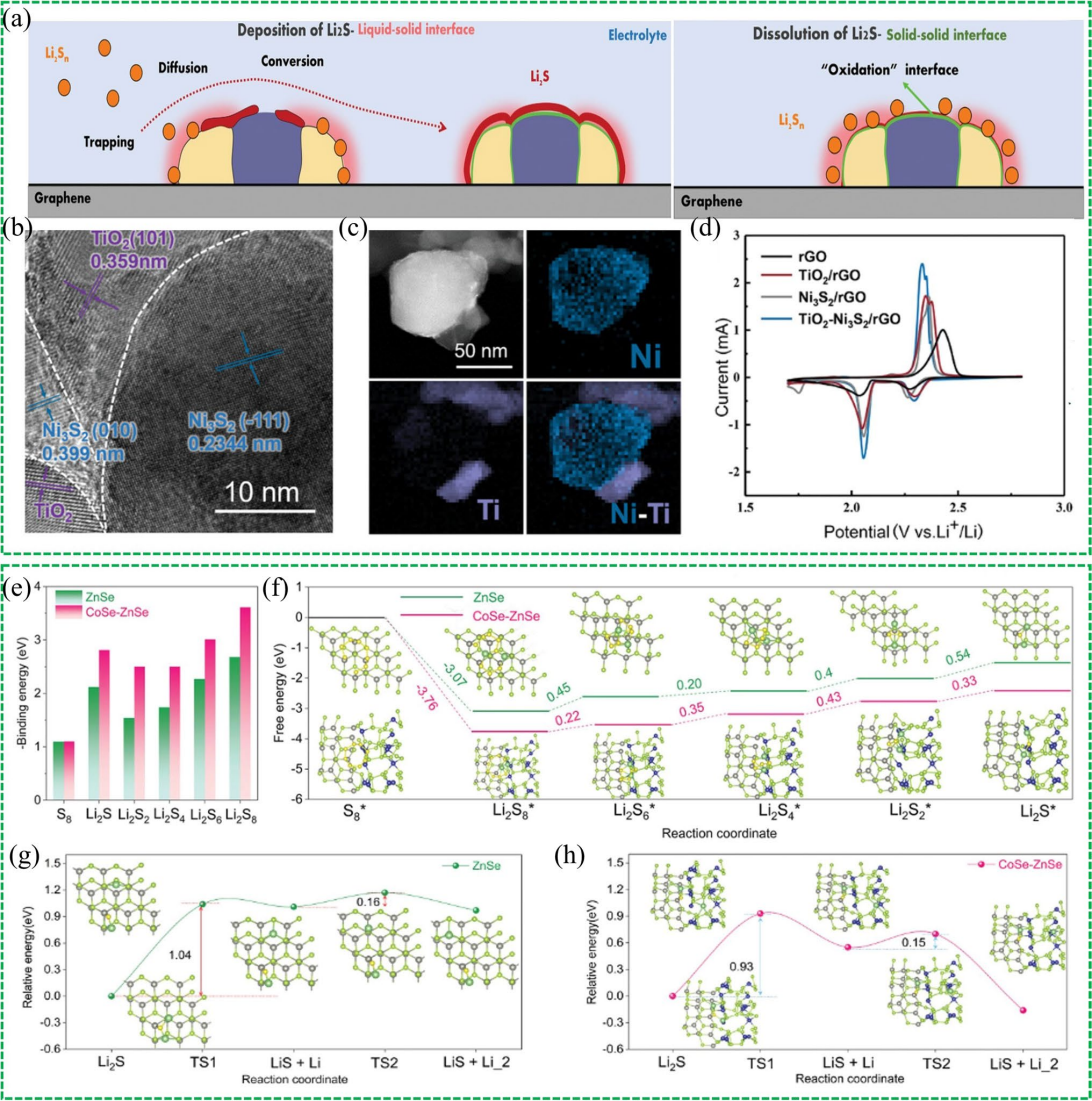
Reproduced with permission from Ref. [105]. Copyright 2020 John Wiley and Sons; **a** Schematic illustration of Li_2_S deposition on the liquid–solid interface and the smooth Li_2_S dissolution on the “oxidative” interface. **b** TEM image and **c** EELS element mappings of TiO_2_–Ni_3_S_2_/rGO. **d** CV curves of four different samples. Reproduced with permission from Ref. [106]. Copyright 2021 John Wiley and Sons; **e** Calculated adsorption energy of sulfur species with ZnSe and CoSe–ZnSe heterostructure. **f** Relative free energy for the reduction S_8_ to Li_2_S on the ZnSe and CoSe–ZnSe heterostructure. Energy profiles of the decomposition barriers of Li_2_S and lithium-ion diffusion on the **g** ZnSe and **h** CoSe–ZnSe heterostructure

To this end, Yang’s group developed the heterostructure of small TiO_2_ nanoparticles decorated on large Ni_3_S_2_ nanoparticles (TiO_2_–Ni_3_S_2_) to promote the reduction and oxidation reactions simultaneously [105]. In this structure, the TiO_2_ trap the polysulfides and Ni_3_S_2_ facilitates the electron transfer, and thus can accelerate the conversion of polysulfides in the SRR. And in the SER, both of them can catalyze the oxidation of Li_2_S/Li_2_S_2_ covered on the surface (shown in Fig. 11a). The heterostructure of TiO_2_–Ni_3_S_2_ was confirmed by SEM and EELS element mappings (Fig. 11b, c), from which the tightly contacted interfaces between TiO_2_ and Ni_3_S_2_ can be observed, which ensures the fast diffusion of trapped LiPSs by TiO_2_ to Ni_3_S_2_ surface to enhance the conversion of S species. The bidirectional catalytic properties of TiO_2_ to Ni_3_S_2_ can be reflected by the CV curves (Fig. 11d), the reduction peak of the Ni_3_S_2_/rGO cathode shows much higher current density and slightly shifts to a higher potential in comparison to that of rGO, indicating the enhanced LiPSs conversion reactions. The oxidation peaks of TiO_2_/rGO and Ni_3_S_2_/rGO cathodes shift to a lower potential, suggesting TiO_2_ and Ni_3_S_2_ possess good oxidation ability toward Li_2_S_2_/Li_2_S. Ye et al. developed a CoSe–ZnSe heterostructure which can bidirectionally boost the reaction kinetics for Li–S batteries [106]. A systematic DFT calculation was conducted to investigate the bidirectional catalytic activity of sulfur conversion on the CoSe–ZnSe heterointerface at the atomic level. The binding energies of LiPSs at six different lithiation stages on CoSe–ZnSe heterointerface is higher than those on ZnSe surface (Fig. 11e), indicating the conversion reactions of S_8_ and Li_2_S are more like to occur on CoSe–ZnSe heterointerface. The energy barrier for the reduction from Li_2_S_8_ to Li_2_S_2_ the reaction from Li_2_S_2_ to Li_2_S for the CoSe–ZnSe are lower than that for ZnSe (Fig. 11f), suggesting the sulfur reduction is thermodynamically more favorable on this interface than on ZnSe. With respect to the oxidation reactions, the overall Li_2_S decomposition includes two steps: a single Li ion dissociates from Li_2_S and the dissociated Li^+^ diffuses away from the LiS cluster. The energy profiles for Li_2_S decomposition processes on ZnSe and CoSe–ZnSe are shown in Fig. 11g, h. The calculated dissociation energy barrier of Li_2_S on CoSe–ZnSe heterostructure (0.93 eV) is lower than that on ZnSe (1.04 eV), indicating the CoSe–ZnSe can accelerate the phase transformation of Li_2_S during charging process. As a result, the 3D CoSe–ZnSe@G, the carbon substrate incorporating fully exposed catalytic CoSe–ZnSe heterointerface, facilitated ionic transport and bidirectional sulfur conversion under high sulfur loading and lean electrolyte. Recently, the p-Co_3_O_4_/n-TiO_2_-HPs [107], 1 T-VS_2_ − MXene [108], TiOxNy-TiO_2_ [109] etc. have been reported as the bidirectional catalysts to enhance the sulfur conversion efficiency.

#### Defect Manipulating and Facet Engineering

**Fig. 12:**
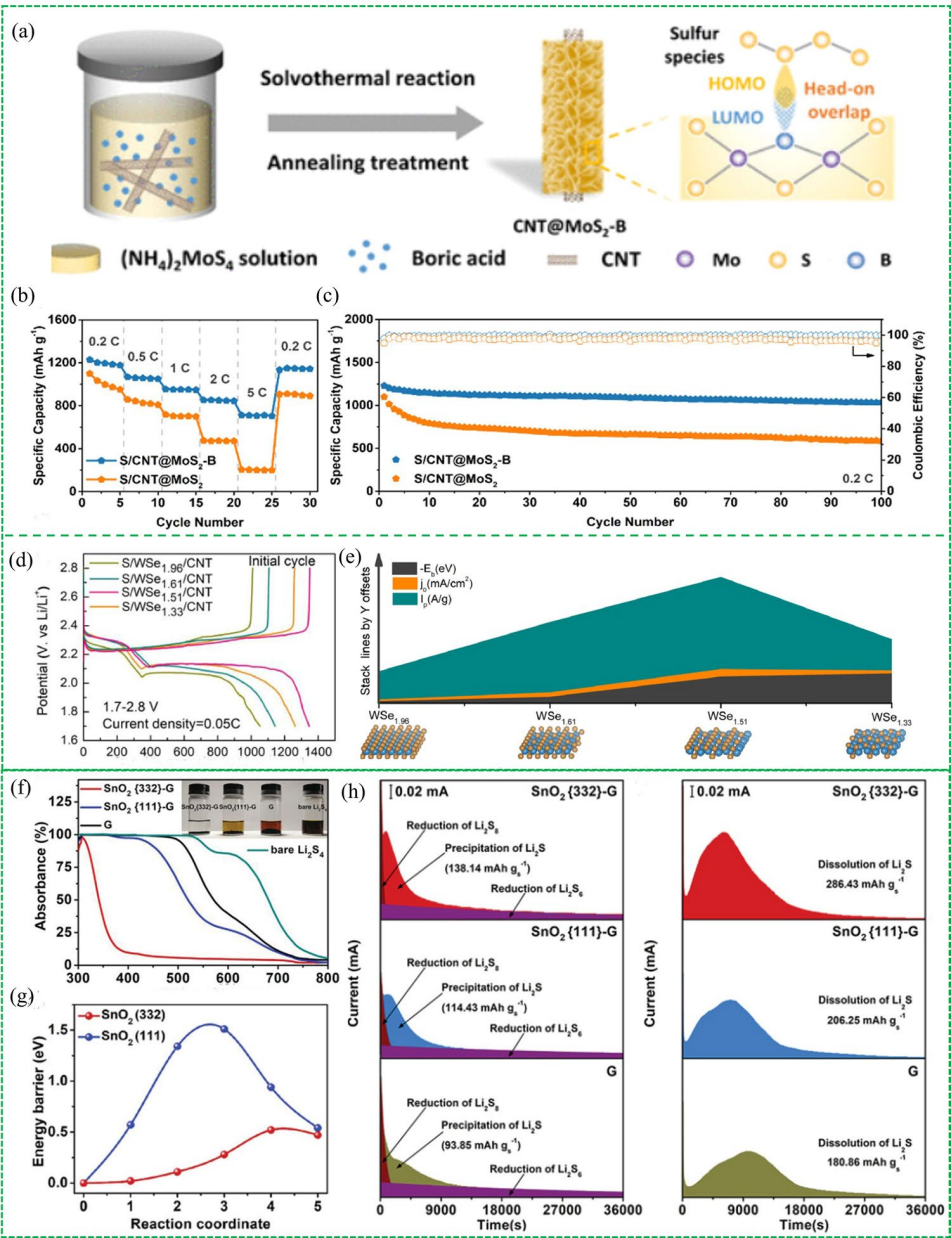
Reproduced with permission from Ref. [110]. Copyright 2021 American Chemical Society; **a** Schematic for the synthesis of CNT@MoS_2_-B. **b** Rate performance and **c** cycling stability of S/CNT@MoS_2_-B cathode and S/CNT@MoS_2_. Reproduced with permission from Ref. [112]. Copyright 2022 Elsevier; **d** Charge/discharge curves of WSe/CNT samples with different vacancies. **e** Electrokinetic performance comparison of electrocatalysts with different Se vacancy. Reproduced with permission from Ref. [113]. Copyright 2021 Elsevier; **f** UV–vis spectrums and optical photographs of a bare Li_2_S_4_ solution and the solutions with SnO_2_(332)-G, SnO_2_(111)-G, G after statical adsorption for 12 h. **g** Decomposition energy barriers of Li_2_S adsorbed on SnO_2_(332) and SnO_2_(111). **h** Potentiastatic discharge profiles of LiS_2_ nucleation and dissolution on three different materials

Defect engineering is regarded as an effective method to optimize the electronic structure and elevate the electrocatalytic activity via adjusting atomic distribution and altering surface properties. Doping and vacancy are two widely-used approaches to enhance the catalytic activity of materials. MoS_2_ has been reported as an attractive catalyst for polysulfide conversion reaction due to its good electrochemical activity, stability and cost-effectiveness. But the active sites are limited since the electrocatalytic activity of MoS_2_ is almost located in the edges rather than basal planes. And the low intrinsic conductivity of the basal hinders the electron transfer for redox reactions. In light of this, Tian et al. reported an orbital engineering strategy to fabricate the B-doped MoS_2_ on carbon nanotubes (CNT@MoS_2_-B) as an electrocatalytic cathode material [110]. B in the B-MoS_2_ is sp^3^ hybridized, and it has a vacant σ orbital perpendicular to the basal plane, allowing for maximal overlap with S species (Fig. 12a). Therefore, the B doping can increase the activated site and enhance electron transfer on the basal planes of MoS_2_. As a result, the CNT@MoS_2_-B host showed excellent rate performance (711 mAh g^–1^at 5C) and super cycling performance with a capacity fading rate of 0.020% in comparison with the CNT@MoS_2_ host (Fig. 12b, c).

Vacancies in various nanocatalysts show efficient catalytic activity, but the vacancy content is positively related to the catalytic properties since excessive vacancies can decrease the electrical conductivity and weaken PS adsorption [111]. To this end, Li et al. proposed a defective WSe_2_ with Se vacancies as a catalytic host material for Li–S batteries and quantitatively investigated the influence of the Se vacancy ratios on the catalytic activity for S conversion [112]. It was proved that the appropriate introduction of Se vacancies is conducive to the catalytic activity of WSe_2_, but the electrocatalytic activity and structural stability of WSe_2_ will decrease with the further increase of Se vacancies. The charge/discharge curves manifest that WSe_1.51_/CNT sulfur cathode exhibits the lowest redox polarization (Fig. 12d). In addition, a series of electrokinetic measurements were conducted to inclose that the WSe_1.51_ with the suitable vacancies possesses the highest electrochemical activity in catalyzing the bidirectional sulfur redox reactions (Fig. 12e).

In addition to component adjustment, morphologic tuning and electronic optimization, the unique electrocatalytic effect of each crystal facet makes difference in the catalytic activity for S conversion in the Li–S realm. Uncovering the catalytic effect of crystal facets is of significant importance to understand the reaction mechanisms and can instruct the rational design of electrocatalysts by facet optimization. Since the crystal facet of nanocatalysts could have selective electrochemical activity for accelerating different steps in Li–S batteries, the rational adjustment of the crystal facet makes it possible to achieve bidirectional enhancement of conversion reactions. For example, Jiang et al. synthesized different SnO_2_ nanocrystals supported on reduced graphene oxide (SnO_2_-G) by regulating the exposed facets and systematically investigated the crystal facet effect in Li–S chemistry [113]. DFT simulation and various measurements were conducted to probe the difference in catalytic ability between SnO_2_ (332) and SnO_2_ (111) facets. The binding energy values of Li_2_S_4_ on the SnO_2_ (332) facet and SnO_2_ (111) are − 5.46 and − 3.07 eV respectively, indicating the SnO_2_ (332) facet exhibits stronger adsorption for Li_2_S_4_ than the SnO_2_ (111) facet. The result was further confirmed by visualized adsorption experiments, in which the Li_2_S_4_ solution with SnO_2_ (332) was transparent while the solution containing SnO_2_ (111) remained yellow after 12 h (Fig. 12f). When it comes to the decomposition reactions of Li_2_S, the calculated decomposition energy barrier of Li_2_S on SnO_2_ (332) is 0.52 eV, lower than that on SnO_2_ (111) facet (1.51 eV), suggesting SnO_2_ (332) crystal facets possess more efficient catalytic activity for Li_2_S decomposition in comparison with SnO_2_ (111) facet (Fig. 12g). Deposition and dissociation processes of Li_2_S were investigated by potentiostatic discharge/charge profiles in Fig. 12h, which showed that SnO_2_(332)-G possess higher Li_2_S nucleation and dissociation capacity than SnO_2_ (111), indicating the SnO_2_(332)-G can enhance the kinetics of both SRR and SER process.

#### Alloying

Metal-based nanoparticles supported on carbon substrates have been shown to be effective catalysts for sulfur conversion due to their high surface free energy and abundance of active sites. However, the weak interaction between these nanoparticles and carbon limits their catalytic performance. To improve catalytic activity, researchers have focused on the alloying strategy, which enables synergistic metal–metal interactions that optimize the electronic structure of individual metals. This approach has been especially effective in releasing the electrocatalytic effect of metals.

**Fig. 13:**
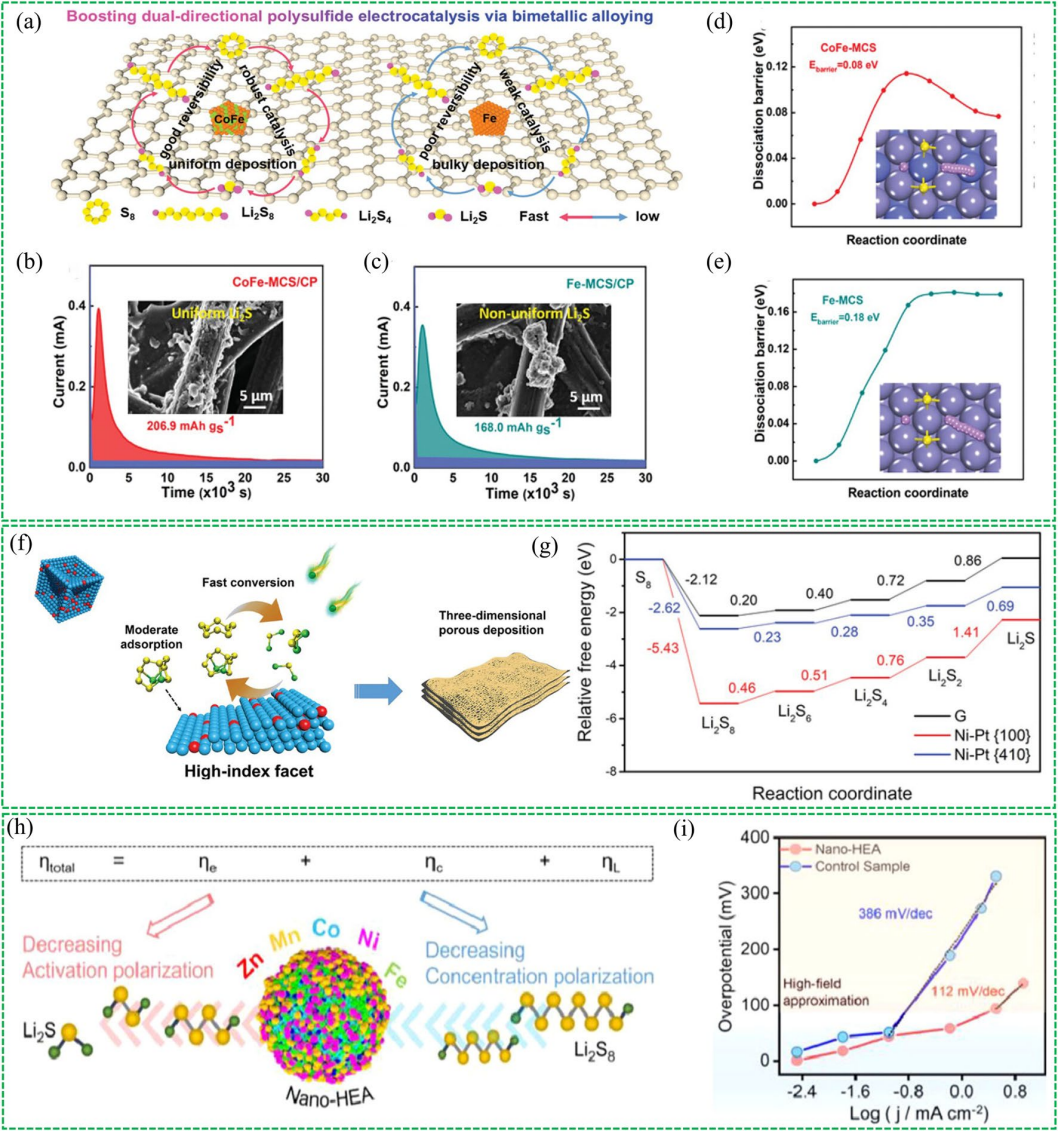
Reproduced with permission from Ref. [114]. Copyright 2020 John Wiley and Sons; **a** Schematic illustration of CoFe alloy cluster supported on carbon substrate to accelerate the bidirectional polysulfide electrocatalysis in comparison with bare Fe cluster. Potentiostatic discharge profiles of Li_2_S_8_ solution on **b** CoFe-MCS/CP and **c** Fe-MCS/CP, respectively. (Inset: SEM images of deposited Li_2_S on two samples). DFT simulation on dissociation barriers and pathways of Li_2_S_2_ cluster on (110) lattice plane of **d** Co_0.3_Fe_0.7_ alloy and **e** Fe matrix. Reproduced with permission from Ref. [115] Copyright 2022 John Wiley and Sons; **f** Schematic illustration of fast conversion of LiPSs and precipitation of Li_2_S_2_/Li_2_S on Ni–Pt {410}. **g** Energy profiles for the sulfur reaction process on three samples. Reproduced with permission from Ref. [116] Copyright 2021 Elsevier; **h** Schematic diagram of the reduced reaction polarization with nano-HEA. **i** Comparison of Tafel slopes with or without the introduction of nano-HEA

Shi et al. proposed the CoFe alloy clusters embedded on carbon spheres (CoFe-MCS) via a bimetal-organic framework pyrolysis strategy [114]. The CoFe bimetal alloy enables strong interaction with polysulfides and electrocatalytic activity toward bidirectional redox reactions in Li–S chemistry (Fig. 13a). The bidirectional electrocatalytic capability of the alloy has been confirmed through detailed characterization, theoretical calculation and in situ instrumental probing. The current response curves of CoFe-MCS and Fe-MCS at 2.05 V are shown in Fig. 13b, c, in which the integrated area corresponds to the capacity of Li_2_S nucleation. The capacity for CoFe-MCS (206.9 mAh g^−1^) is larger than that for Fe-MCS (168 mAh g^−1^), suggesting that CoFe alloy exhibits more efficient Li_2_S nucleation than that of monometal Fe. Additionally, the dissociation pathway and barrier of Li_2_S_2_ on the surfaces of CoFe and Fe were calculated by DFT (Fig. 13d, e). The Co_0.3_Fe_0.7_ (110) surface not only lowers the dissociation reaction energy barrier from 0.18 to 0.08 eV but also reduces its activation barrier from 0.18 to 0.11 eV, indicating that the Co_0.3_Fe_0.7_ alloy possesses higher catalytic activity for Li_2_S_2_ oxidation in the charging process than bare Fe.

The structural effect and catalytic mechanism of alloy electrocatalysts are still unclear in Li–S chemistry and the rational structural design of alloy electrocatalysts remains a challenge. To this end, wang et al. first prepared the well-designed concave-nanocubic Ni-Pt alloy crystallites bounded by HIFs (donated CNC Ni-Pt) on the graphene substrate [115]. The CNC Ni-Pt not only exhibited a moderate chemical anchoring toward LIPSs but also boosted the conversion of intermediate polysulfides (Fig. 13f), which have been confirmed by different measurements. The DFT simulation was performed to investigate the conversion of sulfur on Ni–Pt {410} and Ni–Pt {100} surfaces to probe the high catalytic activity of CNC Ni-Pt. In this simulation, Ni–Pt {410} and Ni–Pt {100} are representatives of high-index facets (HIFs) and low-index facets LIFs, respectively. The first reduction step is a spontaneous reaction and the last step (from Li_2_S_2_ to Li_2_S) is the rate-determining step on the three different surfaces. Compared with Ni–Pt {100} and Ni–Pt {100}, the Ni–Pt {410} deliveries the lowest energy barrier on the formation of Li_2_S_6_, Li_2_S_4_, Li_2_S_2_, and Li_2_S (Fig. 13g), suggesting that the conversion reactions is the most thermodynamically favorable. In addition, the dissociation energy barriers of Li_2_S_6_ on Ni–Pt {100} and Ni–Pt {410} were studied, which showed that the energy requirement of dissociating Li_2_S_6_ on {410} facets is lower than that on {100} facets, indicating the Ni-Pt {410} facets showed higher catalytic activity for bidirectional redox kinetics than Ni–Pt {100} facets. This work underlined the effectiveness of the rational structural design of alloy electrocatalysts for enhancing Li–S chemistry and provided new possibilities for the structural design of highly-effective electrochemical hosts.

High-entropy alloy (HEA), which is usually composed of five or more different metal elements, possesses the inherited merits from each component and synergistic regulation of electronic properties, shows great potential for catalyzing electrochemical reactions. Recently, HEA electrochemical catalysts have attracted more attention for catalyzing redox reactions in Li–S chemistry. Xu et al. developed a nano high-entropy alloy (nano-HEA) consisting of Fe, Co, Ni, Mn, Zn five elements as a holistic electrocatalyst to boost sulfur redox reactions [116]. The nano-HEA can help to reduce reaction polarization and energy loss, which enhances the bidirectional conversion reactions in Li–S chemistry (Fig. 13h). The Tafel slope of this nano-HEA is about 3.5 times lower than that of the sample without the nano-HEA (Fig. 13i), suggesting that the sulfur redox reaction is kinetically favorable on the nano-HEA. DFT calculations illustrated that the catalytic activity of HEA relies on the optimized redistribution of surface charges, and different metallic atoms play unique roles in optimizing surface charges. This work opened a new door for bidirectional electrocatalysis with good durability in Li–S batteries.

#### Single Atom Tailoring

Single atom catalysts (SACs) show great promise for achieving robust electrocatalytic activity and selectivity in Li–S chemistry due to their abundant catalytic sites, high atom utilization and small volume/weight occupancy. Considering various atom candidates, the theoretical modelling-guided strategy can accelerate the search for promising catalytic atoms, thereby increasing the efficiency and accuracy [117, 118]. In this regard, Zhou et al. used DFT to screen various SACs ranging from Fe, Mn, Ru, Zn, Co, and V [119]. Under the guidance of DFT calculations, large-scale single atom vanadium catalysts supported on graphene were prepared to achieve high performance Li–S batteries. It was confirmed by experimental results and theoretical calculations that the single V atom catalyst can not only anchor LiPSs but also catalytically convert LiPSs/Li_2_S during cycling, thereby improving sulfur utilization, rate performance and cycling stability.

**Fig. 14:**
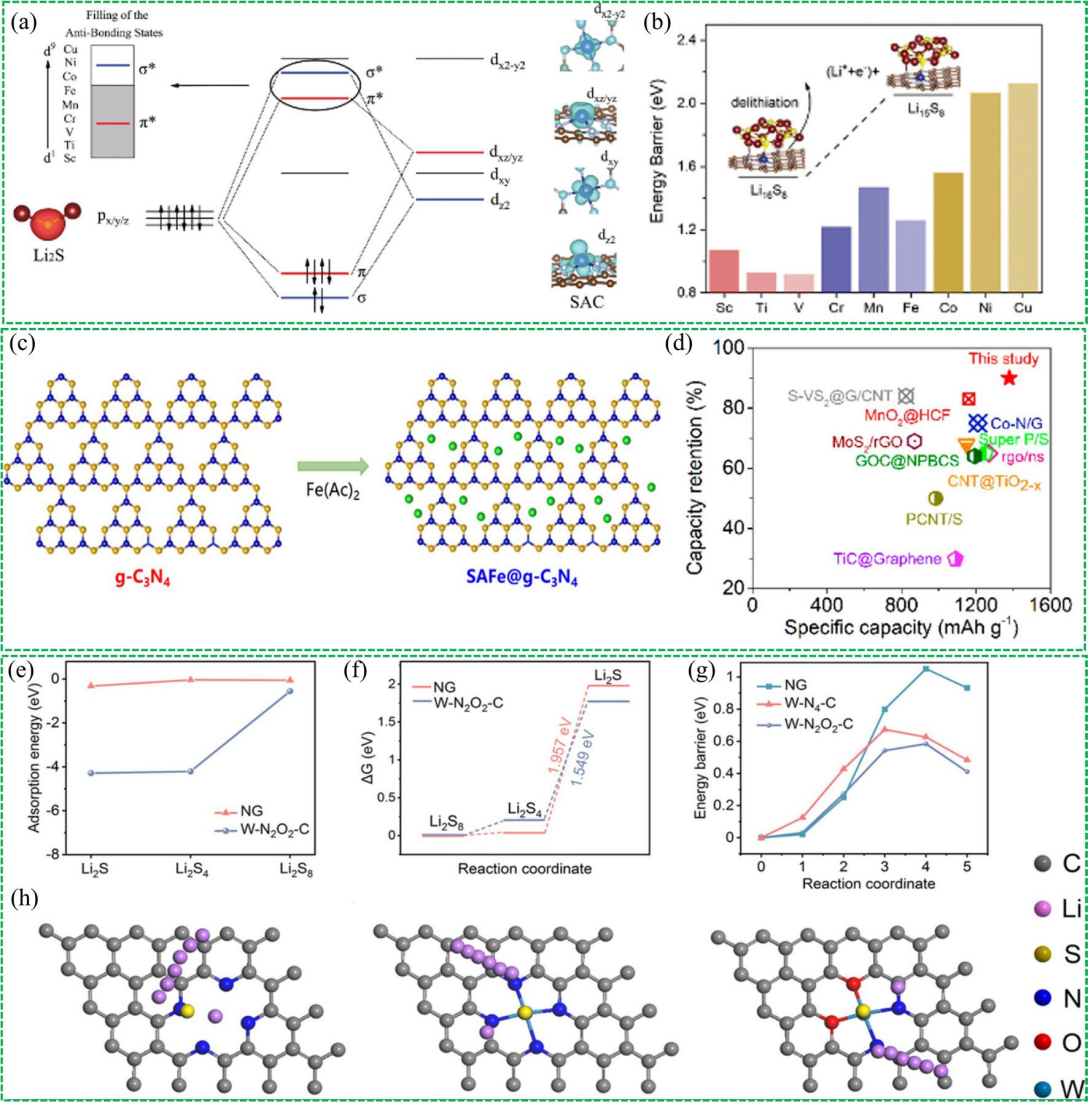
Reproduced with permission from Ref. [120]. Copyright 2021 John Wiley and Sons;** a** d-p orbital hybridization scenario between SAC and Li_2_S. **b** Energy barrier for the oxidation of Li_2_S on different SACs. Reproduced with permission from Ref. [121]. Copyright 2020 American Chemical Society; **c** Schematic illustration of synthesis strategy for SAFe@g-C_3_N_4_. **d** Comparison of various cathode materials of Li–S batteries. Reproduced with permission from Ref. [123]. Copyright 2021 John Wiley and Sons; **e** Adsorption energies of NG and W/NG for Li_2_S_8_, Li_2_S_4_, and Li_2_S. **f** Gibbs free energy changes of Li_2_S_8_, Li_2_S_4_, and Li_2_S on NG and W/NG. **g** Energy barrier profiles for the dissociation of Li_2_S on NG, W-N_4_-C and W-O_2_N_2_-C. **h** Decomposition pathways of Li_2_S on NG, W-N_4_-C and W-O_2_N_2_-C

Additionally, Cheng’s group conducted electronic structure calculations and proposed d-p orbital hybridization between a metal atom and sulfur species can be used to evaluate the catalytic activity of SACs [120]. The evaluations focused on nine types of 3d metals ranging from Sc to Cu, and revealed Ti atom possesses more efficient d-p hybridization and is more likely to form stronger bonds with S due to the slightly filled π* states (Fig. 14a). The strong d-p hybridization in SACs-S bond can not only effectively anchor Li_2_S but also lower the energy barriers to bidirectional redox reactions (Fig. 14b). The relative experiments were subsequently conducted to confirm the calculation by implanting several SACs on 3D carbon fiber foam using controllable N coordination. Among the electrodes, SATi/CF showed the best catalytic properties, well consistent with the theoretical calculations. Due to the higher catalytic property of SATi on the carbon substrate, the SATi/CF-S cathode delivered high specific energy with a low catalyst loading (1 wt%) and a high area-sulfur loading (8 mg cm^−2^). This work elucidated the modulation essence of the d-p orbital hybridization on Li–S chemistry and offered valuable insight for creating highly catalytic SACs for high performance Li–S batteries.

Notably, the metal loading of most reported SACs was very low since higher loading can lead to aggregation of single atoms which can reduce the catalytic activity of SACs. Hence, it is of great importance to explore high-loading single-atom materials to achieve faster conversion of sulfur. To this end, Liu et al. selected g-C_3_N_4_ with abundant N sites as support for a single Fe atom to prepare a high-loading single-atom catalyst (SAFe@g-C_3_N_4_) (8.5 wt%) (Fig. 14c) [121]. The DFT simulation was used to verify that the introduction of SAFe to g-C_3_N_4_ can significantly decrease the energy barrier of dissociation of Li_2_S and thus boost redox kinetics. As a result, the SAFe@g-C_3_N_4_-S cathode exhibited a high specific capacity of 1,379 mAh g^–1^ at 0.1C and 704 mAh g^–1^ at 5C. In addition, the cathode presented excellent cycling stability with a capacity retention of 90% after 200 cycles at 0.2C. In comparison to the specific capacity and capacity retention of various cathode materials for Li–S batteries, the SAFe@g-C_3_N_4_ cathode can compete with most reported materials (Fig. 14d). To further pursue higher single atom content, Liu et al. proposed a salt-template approach to synthesis monodispersed Co single atoms seeded in nitrogen-doped carbon nanosheets with up to 15.3 wt% of Co [122]. This method offered inspiring opportunities for rational design and fabrication of high-loading single atomic catalysts.

In addition to the coordination of atom number, the coordination of atom type is also important to the optimization of the electronic structure of SACs to enhance the electrocatalytic effect. In this regard, Wang et al. fabricated the unique W-O_2_N_2_-C (tungsten single atoms embedded on N-doped graphene) with high W loading of 8.6 wt% via a facile self-template and self-reduction strategy [123]. DFT calculations were performed to probe the origin of the enhanced catalytic activity and adsorptive property of the W-O_2_N_2_-C structure. The adsorption energies of nitrogen-doped graphene (NG) and tungsten (W) SAC immobilized on nitrogen-doped graphene (W/NG) for Li_2_S_8_, Li_2_S_4_, and Li_2_S are shown in Fig. 14e, the W/NG exhibits stronger adsorption ability towards Li_2_S_8_, Li_2_S_4_, and Li_2_S than that of NG. Additionally, the W/NG delivers lower ΔG (1.549 eV) of Li_2_S_4_/Li_2_S conversion (the rate-determining step) than that of NG (1.957 eV), suggesting that the LiPSs conversion is more thermodynamically favorable on W/NG in comparison with NG substrate (Fig. 14f). The dissociation barrier of Li_2_S on NG, W-N_4_-C and W-O_2_N_2_-C surfaces was calculated to reveal the promoted electrocatalytic activity of the unique W-O_2_N_2_-C coordination (Fig. 14g, h). W-N_4_-C exhibits a lower barrier (0.62 eV) for Li_2_S decomposition than NG (1.05 eV), which means the W-N_4_ coordination can accelerate the conversion of Li_2_S. More importantly, when two O atoms are introduced into the W-N_4_ coordination to form W-O_2_N_2_-C, the energy barrier of Li_2_S decomposition on the catalyst further decreases to 0.58 eV, indicating the W-O_2_N_2_ coordination shows higher catalytic property than the W-N_4_. This work revealed the effect of local coordination environment on adsorption and catalytic activity for S species and provided a new idea for delicate design of SACs.

## Machine Learning for Efficient Cathode Discovery

**Fig. 15:**
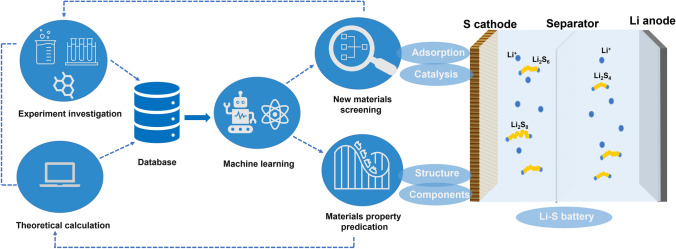
Process of the high-throughput machine-learning-enabled development in battery materials and its application in the discovery of sulfur hosts materials

Recently, with the in-depth research on batteries and the rapid development of computer technology, machine learning (ML) has been developed in battery research to improve efficiency and lower the cost of trials and errors [117, 124]. The general process of the high-throughput machine-learning-enabled development method can be illustrated in Fig. 15, where the database is developed based on experiments and theoretical simulations. Developed machine learning algorithms will be selected according to the database and intended features to implement large-scale data modelling, classification and optimization. Therefore, the new promising candidates with better performance can be screened out and the properties of some materials can be predicted. The screened material can guide experiments to further produce more promising materials while the predicted material properties are beneficial to the modelling in the theoretical calculation and the material designs in the battery materials development process. In the development of Li–S battery cathode materials, the major obstacles of the shuttle effect and sluggish conversion of polysulfides have been focused on. So far, machine learning has been applied to high-throughput screening of new materials with a strong adsorption of LiPSs and catalytic activity. In addition, the material properties including structures and components of the cathodes which are beneficial to the battery performance can be predicted assisted by machine learning.

**Fig. 16:**
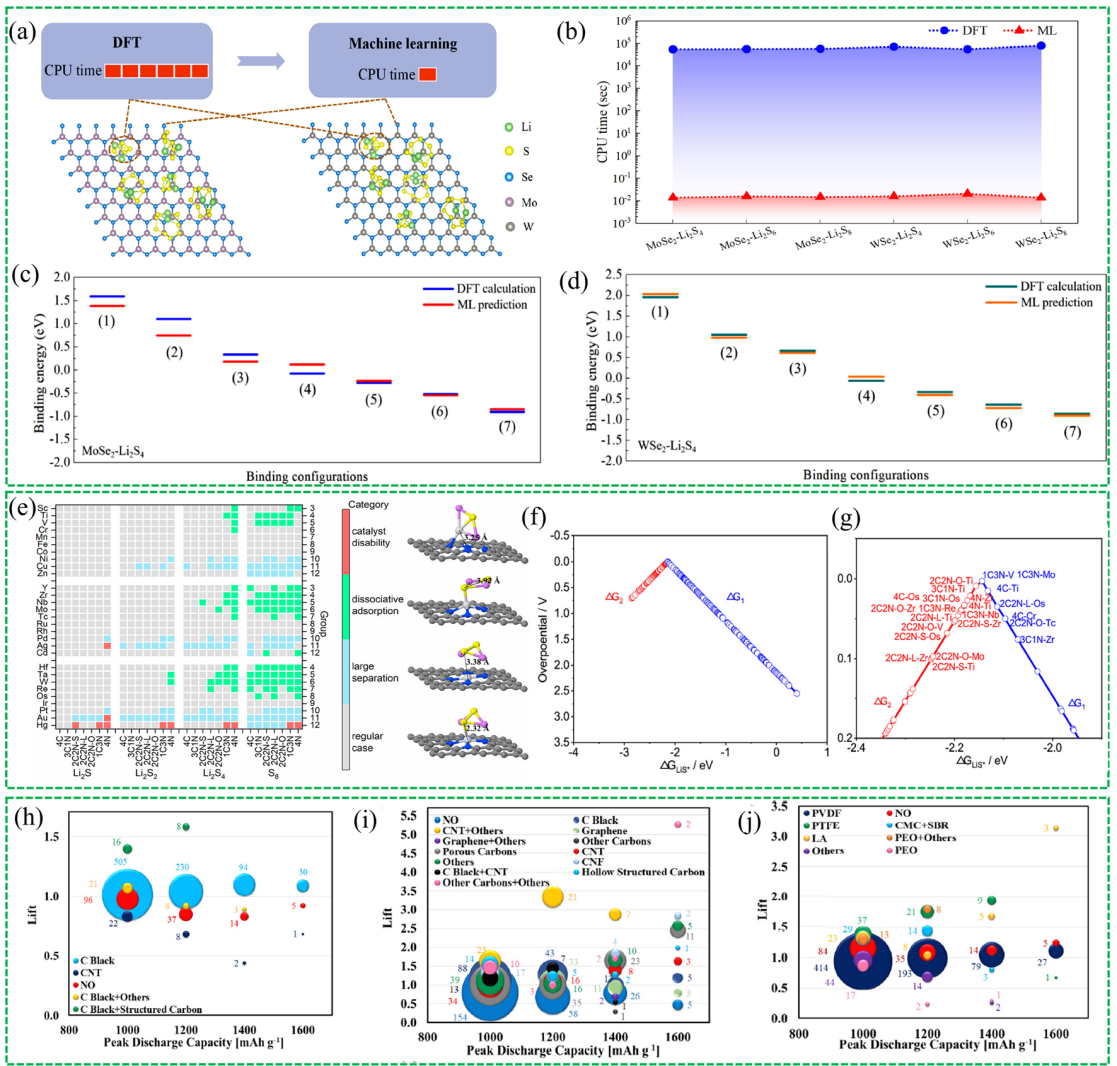
Machine learning method in the screening of adsorptive materials: Reproduced with permission from Ref. [126]. Copyright 2021 Elsevier. **a** Schematic illustration of DFT and ML methods toward adsorption models; **b** Comparison of CPU computational time between DFT and machine learning method for MoSe_2_ /WSe_2_ towards Li_2_S_4_, Li_2_S_6_ and Li_2_S_8_; Binding energy comparison between DFT calculation and ML prediction for **c** MoSe_2_/Li_2_S_4_, **d** WSe_2_/Li_2_S_4_. ML method in the screening of catalytic material: Reproduced with permission from Ref. [127]. Copyright 2021 American Chemical Society; **e** Illustration of categories of adsorption configurations; Predicted adsorption energy of LiPSs and overpotential curves for **f** all catalysts and **g** catalysts with an overpotential lower than 0.1 V. ML method in the assessment and prediction of components of cathode material: Reproduced with permission from Ref. [128]. Copyright 2020 Elsevier; Change of lift with maximum discharge capacity and design factors **h** conductive additive type, **i** encapsulation type and **j** binder type in the cathode of Li–S batteries

To discover shuttle-effect-suppressive sulfur host materials, the conventional experimental identification for potential material is complex. In recent years, high-throughput DFT calculation methods have been developed to investigate the adsorption property to discover promising materials. These methods are normally cost-high and time-exhaust [125]. Hence, based on the development of DFT calculation, machine learning has been introduced to rapidly and accurately predict the binding energies towards LiPSs. For example, Zhang et al. proposed an ML method to research the binding energy of the surfaces of different sulfur hosts towards polysulfides with arbitrary spatial configurations and random sites [126]. Since the layered materials have been used as promising sulfur hosts due to their abundant adsorption sites for LiPSs, the MoSe_2_ and WSe_2_ were selected as the case studies in this work. The three atom layers of MoSe_2_ and WSe_2_ were constructed as substrates for the adsorption of LiPSs (Li_2_S_4_, Li_2_S_6_ and Li_2_S_8_). As illustrated in Fig. 16a, the single-point binding energies between LiPSs and substrates with arbitrary spatial configuration and random sites were first calculated via the DFT method. The data set consisted of thousands of DFT bind energies would be obtained, and then randomly divided into training data and test data. A time-consuming work to get 11,395 binding energies were conducted by DFT calculation, and the scratch (FS) training method would be used to evaluate the binding energies of MoSe_2_ and LiPSs. To accelerate the prediction process, the transfer learning (TL) method was afterwards used to evaluate the binding energies of WSe_2_/ LiPSs with only 1,500 calculated binding energies. Figure 16b compared the CPU computational time of DFT calculation and ML methods for six systems, indicating the speed of the ML method is six orders of magnitude faster than that of the DFT method. In addition, as shown in Fig. 16c, d, the prediction results are very close to the DFT results. Compared with the MoSe_2_-Li_2_S_4_ system, the MoSe_2_-Li_2_S_4_ showed better prediction results, where the predicted binding energies are nearly in line with the DFT data, indicating an excellent accuracy of the prediction model. Based on the model with transfer learning (TL) algorithm, this ML method can apply to other 2D layer host materials with a similar AB_2_ structure to MoSe_2_ and WSe_2_ for predicting their binding energies with LiPSs, thereby discovering promising shuttle-effect-suppressive materials. Subsequently, Zhang et al. proposed an ML method to rapidly discover AB_2_-type sulfur host materials for suppressing the shuttle effect [125]. In this work, 14 new structures (PdN_2_, TaS_2_, PtN_2_, TaSe_2_, AgCl_2_, NbSe_2_, TaTe_2_, AgF_2_, NiN_2_, AuS_2_, TmI_2_, NbTe_2_, NiBi_2_, and AuBr_2_) were discoveried from 1320 AB_2_-type compounds. These structures exhibit strong adsorption for LiPSs and appreciable electron transfer capability, showing great potential in the application of sulfur host materials in Li–S batteries.

The rational design of cathodes with high catalytic activity plays an important role to suppress the problem of sluggish kinetics of sulfur reduction reaction. The doped carbon materials embedded with single-atom catalysts (SACs) have been regarded as a kind of promising sulfur hosts to further boost Li − S battery performance. However, the conventional “trial and error” method or regular DFT calculation is less likely to identify better structures from enormous compositions. To overcome these issues, a machine learning method assisted with high-throughput calculation was proposed by Li’s group to screen SAC on nitrogen-doped graphene [127]. The SAC including 3d, 4d, and 5d elements from the periodic table were investigated. As shown in Fig. 16e, the adsorption configuration was classified into four categories: regular case (C1), large separation (C2), dissociative adsorption (C3) and catalyst disability (C4). The reduction of LiPSs was divided into two reaction steps whose reaction energies were defined as ΔG_1_ and ΔG_2_. The potential limiting step was identified as either ΔG_1_ or ΔG_2_ which depends on the LiS* adsorption energy, exhibiting a volcano curve as shown in Fig. 16f. The result showed the process is limited by LiS* formation from Li_2_S_2_ on 61 catalysts and limited by Li_2_S formation from LiS* on 142 catalysts. The overpotential reaches the minimum value when the adsorption energy is 2.147 eV. According to the screening process, the most promising SAC catalysts were demonstrated in Fig. 16g, the supported V, Mo, Ti, Zr, and Os single-atom catalysts which are on the top of the curve exhibit a very small overpotential. This work not only extends the scope of SAC application but also provides a new strategy for the development of sulfur hosts.

The Li–S battery is a complex system, and the performance of Li–S battery is highly sensitive to the components and cell design. In terms of the cathode, except for numerous sulfur host candidates, a variety of conductive additives and binders have been proven to be beneficial to the improvement in the performance of cathodes. Machine learning is an effective tool to identify the critical connection between the parameters from the vast data, providing instructive guidance for the holistic optimization of cells for practical applications. Kilic et al. analyzed the effect of compositions in electrodes on the performance of a cell from a constructed database containing the data for 1,660 cells through a machine learning method [128]. The results for the Li–S battery cathode design parameters are shown in Fig. 16h–j, where the bubble size represents the number of counts. Figure 16h implies that carbon black, the most popular conductive additive, is still the best choice in comparison to other candidates. The sulfur host types have been assessed in Fig. 16i, which indicates that developing novel porous carbon encapsulation materials is a highly promising way to achieve high discharge capacity in practical applications. The effect of the binder types on the discharge capacity is analyzed in Fig. 16j, where it is seen that PVDF has a constant value at around 1 for all the capacity intervals, indicating it is a more stable binder in these candidates. This study offers valuable insights for the development of practical Li–S batteries through data assessment.

Overall, machine learning is a highly efficient method for screening or predicting the promising sulfur host candidate. Based on some DFT calculation data and models, machine learning can predict the adsorption energy and binder energy between carbon-based hosts and LiPSs for other new candidates with similar structures or properties, significantly increasing the efficiency in discovering promising sulfur hosts in numerous candidates in comparison to the conventional “trial and error” method or regular DFT calculation. In addition, based on the extensive body of research findings, machine learning can establish intricate relationships between diverse parameters, encompassing the types and compositions of binders, conductive additives and carbon substrate structures in cathode electrodes. This approach can lead to significant improvements in the optimization process of developing cathode materials of Li–S batteries. Notably, machine learning has yet deeply penetrated in the development of batteries, largely due to the immaturity of existing models and algorithms, as well as insufficient data. However, it is widely believed that the continued advancement of computer science and battery science will enable machine learning to play a transformative role in the development of batteries.

## Conclusion and Outlook

This review focuses on the advancement of carbon-based materials in achieving high-performance Li–S batteries, given their excellent conductivity, stability, and structural versatility. The review begins by discussing the rational designs of carbon hosts, ranging from 0 to 3D structures, to achieve high sulfur loading and more confinement channels. Furthermore, introducing chemically functional modifications to these materials can further enhance their performance as sulfur hosts. The strategies for chemical modification are comprehensively explored, including chemical adsorption and electrocatalysis. Regarding chemical adsorption, two types of chemical interaction between sulfur hosts and polysulfides are identified: polar-polar and Lewis-acid base interactions. These interactions effectively confine polysulfides through chemical bonding. In terms for electrocatalysis, four strategies to increase the electrocatalytic activity of carbon-based host materials are discussed: heterostructure engineering, defect manipulating and facet engineering, alloy optimization, and single atom tailoring. These strategies enable host materials to effectively anchor intermediate polysulfides and accelerate the redox kinetics during the charge and discharge processes. Finally, the review delves into the machine learning method as an effective way to screen promising materials and analyze vast amounts of data to accelerate the discovery of cathodes for practical Li–S batteries.

Based on the advancement of Li–S batteries, an ideal sulfur host material should possess four properties simultaneously: (1) excellent conductivity and appropriate porosity; (2) strong anchoring ability for dissolved LiPSs; (3) high electrocatalytic activity for bidirectional redox reactions, (4) Heterostructure to minimize the required electrolyte volume. To prepare an ideal host material, the merits of structure design and chemical modification should be comprehensively considered. The simple methods for structure design, which are compatible with the chemically modified strategies, are preferred to prepare porous carbon substrates (3D interconnected hierarchically porous carbon, hollows carbon and MOF-based carbon material) without sacrificing electrical conductivity. The structural design can not only provide more space for chemical modification and achieve high sulfur confinement but also provide more electron transfer pathways to accelerate redox reactions. Regarding chemical adsorption, it should be considered with the catalytic properties to achieve a synergistic effect. The candidates with strong anchoring ability can be integrated with those catalytic compounds to form heterostructure to anchor LiPSs and enhance the conversion reactions. The superior bidirectional electrocatalysts can be optimized by defect or facet engineering and alloy optimization strategies. In addition to heterostructure catalysts, the single-atom catalyst is also a promising alternative due to its abundant catalytic sites, high atom utilization and small volume/weight occupancy. It should be paid more attention to developing SACs via atoms coordination or optimization of electronic structure to achieve high catalytic activity for bidirectional conversion reactions. Notably, based on the advancement of research work, the machine learning method is more likely to play a vital role in the development of practical Li–S batteries. Therefore, the combination of experiments and machine learning is a promising strategy.

## Challenges and Future Directions of Developing Carbon-Based Hosts for Practical Application

While the discussed strategies are effective in optimizing cathode hosts and significant progress has been made in the development of Li–S battery cathodes, obtaining a host material with all-around advantages remains a challenge. Besides the strategies mentioned earlier for preparing an ideal cathode, other factors need to be considered for commercial applications.

*Surface wetting ability:* The inherent characteristics of carbon materials typically exhibit solvophobic behavior, causing poor wetting of carbon-based electrodes by electrolytes and leading to low electrolyte utilization efficiency. Therefore, improving the wettability of carbon-based hosts need to be considered. To address this issue, various modifications can be made to carbon materials, such as heteroatom doping, defect engineering, and functional group binding. Another approach is to combine carbon materials with polar materials that have better infiltration properties with the electrolytes.

*Real energy density*: To improve the cycling stability of Li–S batteries, host materials are often used excessively, which can reduce the volumetric energy density of the battery. Therefore, it is important to increase the sulfur loading of electrodes to achieve satisfactory electrochemical performance while minimizing the use of host materials (more than 7 mg cm^−2^). Additionally, to increase the energy density, the mass of the electrolyte should be reduced, and the cathodes should exhibit good performance under a lean electrolyte condition (E/S ratio < 5 µL mg^−1^). The challenges remain for carbon-based sulfur hosts to balance the surface area and electrolyte mass: porous and functional carbon materials with high surface area possess abundant trapping sites for polysulfides, whilst they consume a large amount of electrolyte due to the pore structure, which limits the energy density for practical Li–S batteries at the device level. The strategy to alleviate this contradiction is to build hierarchical structures to minimize the electrolyte-accessible pores and volumes while remaining the trapping sites for polysulfides. The precise design and calculation of the pore volumes in carbon-based hosts and the aiming sulfur loading would be helpful to improve the pore utilization rate and reduce the excessive electrolytes. When evaluating parameters for Li–S cylindrical or pouch cells, it is recommended to measure and optimize the electrode density as a standard procedure during the production of Li–S batteries.

*Economic feasibility:* Even though the price of sulfur is not high, the preparation of functional sulfur host materials is likely to be costly. Therefore, complex preparation methods should be avoided and the dosage of precious metal precursors to prepare catalysts needs to be controlled.

*Production stability*: To achieve commercial viability, priority should be given to the production stability of proposed sulfur host materials as their complex structures or components make it difficult to consistently produce batches of materials with the same quality, despite these materials demonstrating comprehensively excellent performance.

*Failure mechanism*: With increasing investigation, there has been a growing understanding of the failure mechanism of Li–S batteries and some effective protection strategies have been proposed [129, 130]. However, most of the investigation focuses on coin cells or some in-situ cells, which cannot reflect the real operation condition of practical Li–S batteries, especially under harsh conditions [131]. Currently, it is challenging to figure out the failure mechanisms and regulating strategies for practical Li–S batteries since there is a huge gap between lab-scale coin cells in academic research and device-level pouch cells in practical applications. To narrow this gap, more and more attention has been paid to investigate pouch cell level with high energy density (> 300 Wh kg^−1^) [132]. For instance, Cheng et al. [133] conducted a failure analysis on Li–S pouch cells (300 Wh kg^−1^) under low E/S ratio of 3 µL mgS^−1^ and thin lithium anode of 50 µm. They identified the failure of lithium anode as the main reason for the rapid capacity decay rather than the decomposition of electrolyte. In addition, Shi et al. [134]used patterned electrodes with manipulated surface roughness to make cells under practical conditions, and the test proved that an internal short circuit (ISC) is a root cause of early cell failure, resulting from crosstalk between the S cathode and Li anode. Liu’s group [135] studied the reaction heterogeneity in practical Li–S pouch cells with the energy density of 300 Wh kg^−1^. They proposed the low fluidity of electrolyte is the primary factor leading to the uneven distribution of lithium ions, resulting their preference to deposit in electrolyte-rich regions and exacerbating the lithium metal. Overall, the failure mechanism of practical Li–S batteries is considerably more complex and challenging to investigate than lab-scale cells. Therefore, it is crucial to discover new strategies for investigating practical cells to uncover the failure mechanism, which is a vital factor in developing more reliable Li–S batteries.

*Operational safety:* The formation of lithium dendrite, which can puncture the separator and cause an internal short circuit, is the primary safety concern associated with Li–S batteries [129, 131]. While modifying the Li anode can help address this issue, it's also important to consider the safety concerns of the cathodes. The interaction between electrolyte and sulfur species and the redox reaction between Li anode and S/C cathode, have been demonstrated to induce self-heating and thermal runaway [136]. To further clarify the inducements of thermal runaway for practical Li–S batteries, Jiang et al. [137] systematically evaluated the thermal runaway features of long-term Li–S pouch cells (16 cycles and 45 cycles) with and without additional electrolyte, indicating that the reaction between higher-order polysulfide (Li_2_S_x_ ≥ 6) and Li is the most important trigger of the thermal runaway of cycled Li–S pouch cells. This work uncovers the potential safety risks of Li–S batteries and negative roles of the polysulfide shuttle for Li–S batteries from the safety view.

Hence, to enhance the safety of practical cells, it is essential to consider several factors in terms of cathode materials. Firstly, the sulfur hosts should possess outstanding conductivity and robust adsorption. In addition, the cathode materials should be well-connected to the electrodes to minimize overall ohmic resistance, and the electrodes themselves should have good thermal conductivity. These measures can collectively reduce the risk of lithium dendrite growth and minimize the likelihood of electrolyte and electrode breakdown.

## Will Carbon-Based Hosts Win This Race?

Recently, some intention has been paid to some new promising 2D materials due to the solvophobic property of carbon-based materials. For instance, the pre-lithiated metallic 1 T phase two-dimensional (2D) molybdenum disulfide (LixMoS_2_) was developed to serve as sulfur host, showing strong adsorption of LiPSs, enhanced Li-ion transfer and super electrocatalytic activity for LiPSs [146]. These properties enabled pouch cells to deliver a highly competitive energy density of 441 Wh kg^−1^ and 735 Wh l^−1^. However, the potential of carbon-based hosts should not be overlooked. Significant progress has been made in the development of carbon-based sulfur hosts for the practical implementation of Li–S batteries, with a focus on optimizing cathode structure design and enhancing sulfur redox kinetics. For instance, Chen and co-workers [143] introduced a modular assembly method to fabricate oval-like carbon microstructures (OLCMs) as cathode skeletons which can accommodate volume expansion and facilitate fast ion transportation. As a result, an 18.6 Ah Li–S pouch cell with an impressive energy density of 460 Wh kg^−1^ was achieved, demonstrating the feasibility of achieving high energy density through cathode structure design. Li et al. [140] implanted single-atom N_2_-Fe-B_2_ catalytic sites in carbon hosts and fabricated a 359 Wh kg^−1^ pouch cell. Due to the structure design and efficient catalytic sites, this pouch cell exhibited excellent stability with 92.2% capacity retention after 40 cycles. Li et al. [142] used the modified carbon nanotubes to construct a pouch cell that achieved the highest energy density of 695 Wh kg^−1^. This work represents a breakthrough in fabricating practical Li–S batteries although the fabricated pouch cell delivered poor cycling stability.

The recent advancement of Li–S batteries for practical application can be reflected in Table 3. Although there are still challenges in achieving the practical application of Li–S batteries with high sulfur loading and lean electrolyte and, the benefits of carbon-based hosts outweigh the drawbacks. In addition to the advancement in the carbon-based sulfur hosts for practical application, we believe that carbon-based hosts are still the most promising option for the industrialization of Li–S batteries for some reasons as follow:

1. Carbon is the lightest element in the periodic table that could form a stable conductive simple substance under ambient temperature and pressure. When we design a Li–S battery with high energy density, the ratio for Sulfur/Host should be as high as possible. The carbon-based hosts provide the best chance to achieve a high ratio of Sulfur/Host since it is more likely to achieve high sulfur loading in carbon-based materials via feasible structure design.
2. Carbon material hosts are essential for high-performance Li–S batteries due to their excellent conductivity, structural flexibility, and chemical stability. The high conductivity compensates for the insulating nature of sulfur, while the structural flexibility provides a buffer for the volume expansion of sulfur during charging. Additionally, the chemical stability of carbon material hosts prevents any side reactions with the electrolytes.
3. Carbon-based materials possess inherent structural flexibility, enabling them to be fabricated into different structures to meet specific requirements, thereby increasing their potential to overcome different challenges. Furthermore, carbon materials are highly adaptable and can be modified by combining other functional nanoparticles or groups, which can effectively and simultaneously resolve multiple issues in complex Li–S chemistry.
4. The carbon-based materials have been employed in other batteries (e.g., LIBs) already, and the mature technology, equipment, talented people and industrial chains are ready to transfer once the challenges in Li–S batteries are overcome.

**Table 3:** Comparison of various cathode materials for Li–S pouch cells with over 300 Wh kg^−1^ for practical applications

Sulfur hosts	Sulfur loading (mg cm^−2^)	E/S ratio (µL mg^−1^)	Energy density (Wh kg^−1^) (Rate)	Capacity retention (cycles and rate)	Refs.
Interconnected Carbon Fabric	2.8	2.7	315.98 (0.1C)	81.3% (51 cycles at 0.1C)	[138]
Porous carbon nanotubes	5.6	4	350 (0.05C)	≈90% (40 cycles at 0.05C)	[139]
Fe SAs@B, N-rich carbon matrix	11.6	3 ·	359 (0.1C)	92.2% (40 cycles at 0.1C)	[140]
Modified Ketjent black	3.0	3.5	350 (0.05C)	80% (30 cycles at 0.2C)	[141]
Modified multi-walled carbon nanotubes	7.4	1.7	695 (0.0035C)	62.6% (3 cycles)	[142]
oval-like microporous carbon	8.9	2.7	460 (0.05C)	> 90% (7 cycles at 0.05C)	[143]
ZnS/Co–N–C SAs microporous Carbon	6	4	317 (0.1C)	74% (80 cycles at 0.05C)	[9]
Reactive-type polymer tubes (PQT)	10	3.4	329 (0.1C)	67% (50 cycles at 0.1C)	[144]
Pre-lithiated Mo_6_S_8_	10	1.2	366 (0.05C)	≈90% (10 cycles at 0.1C)	[145]
Pre-lithiated metallic 1 T MoS_2_	7.5	2.4	441 (0.2C)	85% (200 cycles at 0.2C)	[146]
